# Toward AI-Assisted Precision Diagnostics in Breast Cancer: Source-Group-Preserving Evaluation of Class-Imbalance Strategies for Ordered ER-IHC Segmentation

**DOI:** 10.3390/diagnostics16172752

**Published:** 2026-08-27

**Authors:** Md Saiful Arefin, Mohammad Saiful Islam, Md Serajun Nabi, Rashadul Islam Sumon, Maria Lapina, Vitalii Lapin, Mohammed Muthanna

**Affiliations:** 1 School of Computer Science and Informatics, Department of Computer Science, Albukhary International University, Alor Setar 05200, Kedah, Malaysia; mohammadsaifularefin@gmail.com (M.S.A.); saiful.238161@gmail.com (M.S.I.); 2 Faculty of Artificial Intelligence and Engineering, Multimedia University, Cyberjaya 63100, Selangor, Malaysia; md.serajun.nabi@student.mmu.edu.my; 3 Department of Digital Anti-Aging Healthcare, Inje University, Gimhae 50834, Republic of Korea; sumon39.cst@gmail.com; 4 Trusted AI Research Center, Ivannikov Institute for System Programming of the Russian Academy of Sciences, 109004 Moscow, Russia; mlapina@ncfu.ru; 5 Department of Computational Mathematics and Cybernetics, North-Caucasus Federal University, 355017 Stavropol, Russia; vitlx@yandex.ru; 6 Faculty of Computing and IT, Sohar University, Sohar 311, Oman

**Keywords:** ER-IHC, computational pathology, semantic segmentation, class imbalance, nested cross-validation, adaptive minority curriculum, Focal–Tversky loss

## Abstract

**Background/Objectives:** Estrogen receptor immunohistochemistry (ER-IHC) exhibits heterogeneous staining and substantial class imbalance, complicating segmentation of ordered expression categories. We evaluated whether class weighting, minority-focused crop sampling, adaptive minority curriculum (AMC), and Focal–Tversky optimization improved a common ResUNet-DS backbone for segmenting background/non-target pixels and C1 ER-negative, C2 weak-positive, C3 moderate-positive, and C4 strong-positive foreground categories. **Methods:** The dataset comprised 220 paired 512×512 image–mask patches organized into 44 recovered five-patch source groups. A source-group-preserving nested five-fold design used outer folds of 45, 45, 45, 45, and 40 patches. Six controlled training conditions were compared, with four independently selected inner models ensembled for each condition and outer fold. **Results:** Unweighted random training achieved the best numerical mean for all five primary endpoints: foreground Dice (0.7479±0.0123), C2–C4 Dice (0.7098±0.0128), foreground IoU (0.6074±0.0147), foreground quadratically weighted kappa (0.9761±0.0040), and foreground-ordinal MAE (0.0446±0.0062). Weighted random training was the strongest weighted/minority-sensitive condition but did not exceed the unweighted reference. None of 25 paired outer-fold comparisons reached p<0.05; the minimum raw *p*-value was 0.0625, and all Holm-adjusted *p*-values were 0.3125. A 20,000-replicate paired source-group bootstrap preserved the same overall ordering. **Conclusions:** More complex imbalance-handling strategies did not improve aggregate segmentation over unweighted training. Post hoc analyses showed scope-dependent probability quality and error ranking. IHC4BC provided expression-ordering consistency but not direct external segmentation validation. The findings support preliminary source-group-preserving methodological evidence rather than patient-level, whole-slide, or clinical validity.

## 1. Introduction

Estrogen receptor immunohistochemistry (ER-IHC) is an established component of breast cancer pathology because the extent and intensity of nuclear ER staining contribute to tumor characterization and treatment-related assessment. In routine practice, ER-IHC is interpreted visually, and assessment may be complicated by heterogeneous staining, tissue complexity, weakly stained nuclei, and regional variation. Computational pathology therefore offers a means of complementing visual assessment with reproducible spatial maps and quantitative summaries that can support subsequent pathological review [1,2].

Most computational work on breast cancer immunohistochemistry has focused on tumor-region detection, nuclear analysis, image- or slide-level biomarker estimation, or direct scoring. Deep learning has also been investigated for invasive-carcinoma segmentation and supervised segmentation of IHC-stained breast tissue [3,4]. Ordered pixel-level mapping of multiple ER-IHC staining categories is a related but distinct problem: rather than producing a single slide-level score, it preserves where different expression categories occur within an analyzed image region. Such maps may provide an intermediate representation of spatial expression heterogeneity, but they should not be interpreted automatically as clinical ER scores or treatment-decision variables.

The segmentation task considered here contains five labels: background/non-target and four ordered foreground categories, C1–C4. Provenance and mask-semantics auditing identified the foreground classes as C1 ER-negative, C2 weak-positive, C3 moderate-positive, and C4 strong-positive. The ordering is relevant because a confusion between adjacent expression categories differs from an error spanning multiple categories. At the same time, the task is strongly imbalanced: C1 accounts for most labelled foreground pixels, whereas the positive-intensity categories C2–C4 are considerably sparser and may be absent from individual patches. Consequently, a model can obtain favorable aggregate overlap while still performing less consistently on the less-prevalent expression categories.

Class imbalance can be addressed through several mechanisms that do not necessarily have equivalent effects. Loss weighting changes the relative contribution of classes to the optimization objective, minority-focused sampling changes their frequency of presentation during training, and Focal- or Tversky-based objectives alter the penalty assigned to difficult foreground errors [5]. Increasing the complexity of the training strategy, however, does not guarantee better segmentation or better probability behavior. This motivates a controlled comparison in which class weighting, crop selection, adaptive sampling, and Focal–Tversky optimization are varied while the segmentation architecture is held fixed.

Adaptive minority curriculum (AMC) was examined in this context as a training-only crop-selection strategy. AMC modifies exposure to the under-represented C2–C4 categories according to performance observed within the development data, whereas Focal–Tversky modifies the training objective. Neither mechanism changes the inference graph of the common ResUNet-DS backbone. The central methodological question is therefore not whether AMC-Focal constitutes a new architecture, but whether the added complexity of weighting and minority-sensitive training provides reproducible benefit over simpler training under a controlled evaluation.

Reliable comparison also requires attention to dependence among image patches. A provenance audit of the 220-patch resource recovered 44 five-patch source groups. Because patches from the same recovered group may share source-specific visual characteristics, allowing them to cross training, validation, and test partitions would provide an optimistic estimate of generalization. The final evaluation therefore kept each recovered-source group intact within a source-group-preserving nested five-fold design. Inner partitions were used for adaptive-sampling updates and checkpoint selection, while the corresponding outer-test groups remained untouched until final evaluation. The term “source group” is used deliberately because the recovered grouping structure does not independently establish patient, specimen, or whole-slide identity.

The principal endpoints were segmentation overlap and foreground-restricted ordinal agreement with the supplied reference masks. These endpoints quantify performance on the spatial annotation task itself. They do not constitute Allred scoring, H-score estimation, percentage-positive tumor-nucleus measurement, slide-level ER-status classification, pathologist agreement, or treatment-response prediction. Calibration, predicted foreground composition, class-specific probability behavior, predictive entropy, and ensemble disagreement were analyzed separately from frozen outer-test predictions to characterize additional aspects of model behavior without redefining the primary segmentation endpoint. Because pixels within a patch and patches within a recovered-source group are dependent, inferential support was based on paired outer-fold comparisons and source-group-resampling analyses rather than treating individual pixels as independent observations.

Independent external five-class segmentation validation would require a biologically separate ER-IHC cohort with reference annotations compatible with the C1–C4 scheme. Such a cohort was not available. Accordingly, the IHC4BC analysis is retained only as an expression-ordering consistency analysis [6], while the HER2-SISH40x experiment is treated as an exploratory cross-marker and cross-stain stress test [7]. Neither is used as evidence of direct external ER-IHC segmentation accuracy or clinical validity.

Within these boundaries, the study makes four contributions:

1. It establishes a source-group-preserving nested evaluation for the 220-patch ER-IHC resource after recovery of 44 five-patch source groups, preventing recovered groups from crossing training, validation, and outer-test partitions.
2. It compares six training conditions under the same ResUNet-DS inference architecture, including an unweighted reference, class weighting, fixed-minority sampling, AMC, and Focal–Tversky combinations, thereby testing whether additional imbalance-handling complexity is supported by the data.
3. It evaluates the ordered five-class task using segmentation and foreground-ordinal endpoints and supplements these with dependence-aware analyses of probability quality, prevalence distortion, class-specific calibration, and uncertainty-based error ranking derived from frozen outer-test predictions.
4. It defines the evidence boundary explicitly: the work is a preliminary single-resource methodological evaluation of source-group-preserving patch segmentation and does not claim patient-level, whole-slide, independently externally validated, or clinical performance.

## 2. Related Work

### 2.1. AI-Assisted Digital Pathology for Breast Cancer Biomarker Assessment

Whole-slide imaging has enabled computational analysis of tissue morphology, biomarker expression, and spatial heterogeneity in breast cancer pathology. Deep learning has been applied to tumor detection, grading, nuclear analysis, tissue segmentation, and biomarker assessment [8,9,10,11]. These applications are particularly relevant to immunohistochemical biomarkers such as estrogen receptor, progesterone receptor, HER2, and Ki-67, for which interpretation can be affected by staining heterogeneity, spatial variation, and observer-dependent assessment.

Several studies have addressed automated analysis of breast cancer immunohistochemistry. Fauzi et al. investigated automated Allred scoring of ER-IHC whole-slide images [1]. Priego-Torres et al. proposed a deep learning instance-segmentation framework for automated IHC quantification [2]. Liu et al. studied segmentation of invasive-carcinoma regions in breast cancer IHC whole-slide images [3], while Li et al. introduced IHC-IS4BC as a supervised segmentation resource for IHC-stained breast cancer slides [4].

Much of the existing literature therefore targets tumor-region segmentation, nuclear analysis, image- or slide-level classification, or direct biomarker scoring. Ordered pixel-level mapping of multiple ER-IHC staining categories represents a different intermediate task: it preserves spatial information about heterogeneous expression rather than collapsing an image region directly into a single clinical score. Public resources with detailed IHC pixel annotations remain limited, and available datasets often differ in biomarker, staining protocol, annotation granularity, or endpoint definition [12]. These differences complicate both method comparison and direct external validation.

### 2.2. Medical Image Segmentation for Biomarker Mapping

U-Net-style encoder–decoder architectures remain widely used in medical image segmentation because skip connections combine high-level semantic information with fine spatial detail. The nnU-Net framework further demonstrated that strong biomedical segmentation performance can arise from systematic optimization of preprocessing, architecture configuration, training, and post-processing rather than from architectural novelty alone [13]. Residual blocks, feature recalibration, attention mechanisms, and deep supervision have also been incorporated into encoder–decoder networks to facilitate optimization and multi-scale feature representation.

Hybrid convolutional and transformer-based approaches extend this design space by combining local feature extraction with longer-range context modelling. TransUNet introduced transformer components within a U-Net-like framework [14], and subsequent reviews have examined self-attention and CNN–transformer hybrids across medical imaging tasks [15]. General-purpose foundation segmentation models, including the Segment Anything Model, have also been evaluated in medical applications [16,17,18,19,20]. Their transfer to specialized pathology tasks remains challenging because medical images contain modality-specific textures, small structures, ambiguous boundaries, and annotation conventions that differ substantially from natural-image segmentation.

For imbalanced biomarker mapping, architecture is only one component of the problem. A network may obtain favorable average overlap while performing less consistently on sparse expression categories. Accordingly, backbone design must be considered together with crop sampling, loss formulation, class-specific evaluation, and the structure of the validation protocol.

### 2.3. Class Imbalance in Segmentation

Class imbalance can be addressed at data, algorithm, and decision levels. Data-level approaches include oversampling, undersampling, and synthetic minority generation. Algorithm-level methods include class weighting, cost-sensitive learning, and imbalance-sensitive objective functions, whereas decision-level approaches include threshold adjustment and probability recalibration [21,22,23,24].

These mechanisms influence learning in different ways. Resampling changes how frequently observations or image regions are presented during optimization, whereas class weighting changes their relative contribution to the loss. Decision-level adjustment changes the final prediction rule without necessarily modifying the learned representation. Consequently, improvement in a minority-class overlap metric does not necessarily imply improvement in global segmentation, probability quality, or generalization.

In semantic segmentation, imbalance occurs at both image and pixel levels because clinically or biologically relevant structures may occupy only a small fraction of an image. Random crop selection can therefore under-sample rare structures even when a weighted objective is used. Common approaches include weighted cross-entropy, Dice-based losses, Focal loss, Tversky loss, and combinations of distribution- and region-sensitive objectives [5,25].

Sampling and weighting may also alter predicted probability behavior. Evidence from probabilistic clinical prediction has shown that class rebalancing can affect calibration even when discrimination improves [26]. Although dense multiclass segmentation differs from conventional patient-level prediction, the underlying principle remains relevant: hard-label accuracy and probability behavior should be evaluated separately. Neural networks may remain miscalibrated despite favorable predictive performance, particularly when a dominant class strongly influences pooled metrics [27].

For ER-IHC segmentation, this motivates evaluating class weighting, minority-focused sampling, adaptive sampling, and imbalance-sensitive losses under a common architecture rather than assuming that additional imbalance handling is inherently beneficial.

### 2.4. Ordinal Learning for Ordered Expression Categories

Many medical labels, including disease grades, severity categories, and staining-intensity levels, possess an intrinsic order. Treating such labels as unrelated nominal classes ignores the distinction between adjacent and distant errors. Ordinal-learning approaches have therefore been investigated in medical imaging to incorporate label order during training or evaluation [28,29,30].

Most ordinal-learning work has focused on image-level classification rather than dense semantic segmentation. Nevertheless, ordinal evaluation is relevant when foreground classes represent ordered staining categories. In the ER-IHC task considered here, the foreground classes correspond to C1 ER-negative, C2 weak-positive, C3 moderate-positive, and C4 strong-positive. A C2-to-C3 confusion is therefore different in ordinal distance from a C1-to-C4 confusion.

Ordinal-agreement metrics can capture this structure without assuming that the pixel-level classes are equivalent to established clinical scoring systems. In particular, weighted kappa and ordinal mean absolute error provide complementary information to Dice and IoU by penalizing larger class-distance errors more strongly. This distinction is important because ordered spatial annotation performance and clinical scores such as Allred or H-score are related conceptually but are not interchangeable endpoints.

### 2.5. Uncertainty and Calibration in Segmentation Evaluation

Uncertainty estimation and probability calibration describe different properties of predictive models. Predictive uncertainty characterizes dispersion or disagreement in model outputs, whereas calibration concerns the correspondence between predicted probabilities and observed event frequencies. Both have been investigated extensively in medical imaging and model-failure analysis [31,32,33,34].

These issues are particularly relevant in digital pathology because predictions can be affected by staining variation, tissue heterogeneity, scanner differences, ambiguous boundaries, and annotation variability. Uncertainty-based approaches have therefore been investigated as tools for identifying difficult or potentially unreliable histopathology predictions [35]. However, uncertainty and correctness are not equivalent: high uncertainty does not identify every error, and low uncertainty does not guarantee a correct prediction.

Predictive entropy summarizes dispersion in the class-probability distribution, while ensemble variation ratio measures disagreement among hard predictions from multiple models. Error-ranking AUROC can then quantify how effectively an uncertainty score ranks incorrect pixels above correct pixels. This is distinct from calibration: a model may rank errors effectively while its predicted probabilities remain poorly calibrated, or conversely exhibit favorable calibration without strong error-ranking ability.

Calibration itself can also be assessed at different scopes. In dense segmentation, all-pixel measures may be dominated by background, whereas foreground-restricted or class-conditional analyses reveal different behavior. Proper scoring rules such as negative log-likelihood and Brier score quantify probability quality, while bin-based measures such as expected calibration error provide complementary but bin-dependent summaries. These distinctions are important when imbalance-handling strategies alter the effective class distribution encountered during training.

Taken together, prior work motivates an evaluation framework that separates segmentation overlap, ordinal agreement, probability quality, prevalence preservation, and uncertainty-based error ranking. It also motivates accounting for dependence among related image samples rather than relying solely on pooled pixel-level summaries.

## 3. Materials and Methods

### 3.1. Study Workflow

**Figure 1:**
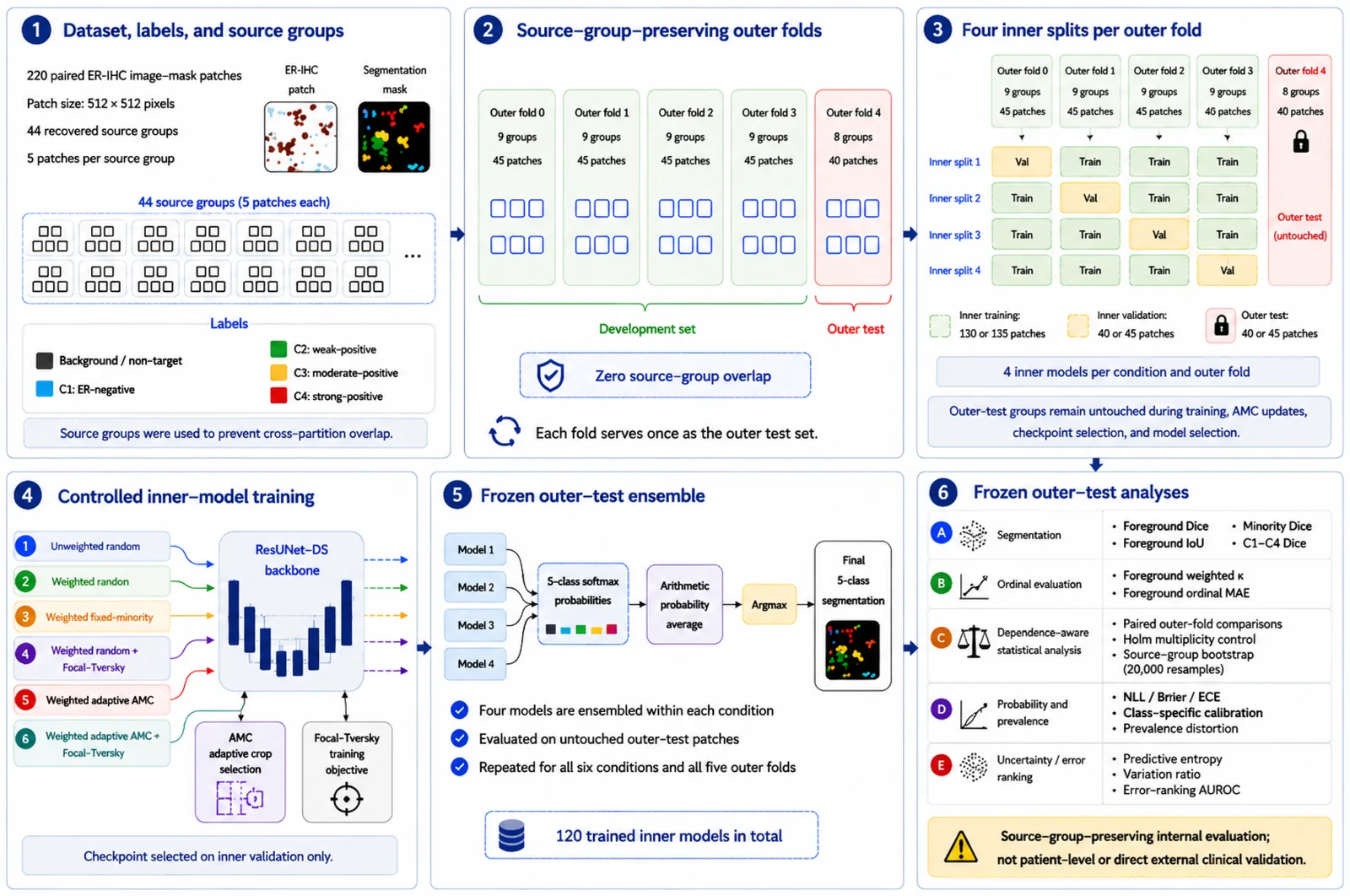
Source-group-preserving nested evaluation workflow for ER-IHC segmentation. The 220 paired patches were organized into 44 recovered five-patch source groups and assigned intact to five outer folds containing 9, 9, 9, 9, and 8 groups (45, 45, 45, 45, and 40 patches). Within each outer iteration, the four development folds rotated once as inner validation, producing four selected inner models per training condition. Their five-class SoftMax probabilities were averaged for evaluation on the untouched outer-test fold. Final analyses were derived from the frozen outer-test predictions using dependence-aware paired and source-group-resampling procedures.

Figure 1 summarizes the final study workflow. The analysis began with quality control of 220 paired ER-IHC image–mask patches and conversion of the supplied color-coded masks to five-class label maps. Provenance analysis recovered 44 source groups, each containing five patches. These recovered groups were treated as indivisible units during partitioning so that patches from the same source group did not cross training, validation, and test partitions.

The 44 source groups were assigned to five outer folds containing 9, 9, 9, 9, and 8 groups, corresponding to 45, 45, 45, 45, and 40 patches, respectively. In each outer iteration, one grouped fold was held out as the untouched outer-test partition and the remaining four folds formed the development set. The outer-test groups were not used for model training, adaptive-sampling updates, checkpoint selection, hyperparameter selection, or post-test model selection.

Within each outer iteration, the four development folds were rotated once each as inner validation. Depending on the sizes of the grouped folds involved, an inner split contained either 130 or 135 training patches and 40 or 45 validation patches, while the corresponding outer-test partition contained 40 or 45 patches. This produced four inner models for every combination of outer fold and training condition.

Six controlled training conditions were evaluated using the same ResUNet-DS inference architecture: unweighted random training, weighted random training, weighted fixed-minority sampling, weighted random training with Focal–Tversky, weighted adaptive minority curriculum (AMC), and weighted adaptive AMC with Focal–Tversky. AMC updates, where applicable, and checkpoint selection were confined to the relevant inner-training–validation split.

After completion of inner-model development, the four selected models for a given condition and outer fold generated five-class SoftMax probability maps for the untouched outer-test patches. The four probability maps were averaged arithmetically, and the final class at each pixel was assigned by argmax. Repeating this procedure across six conditions and five outer folds yielded 120 trained inner models in total.

The frozen outer-test predictions were used for the final segmentation and foreground-ordinal evaluation. Probability quality, predicted foreground composition, class-specific calibration, predictive entropy, ensemble disagreement, and uncertainty-based error ranking were subsequently derived from the same frozen predictions. Statistical support was based on paired outer-fold comparisons and source-group-resampling analyses rather than treating individual pixels as independent inferential observations. Detailed metric and statistical definitions are provided in the corresponding subsections below.

The recovered grouping structure was used to prevent cross-partition source-group overlap; however, the recovered group identifiers are not interpreted as independently verified patient, specimen, or whole-slide identifiers. Accordingly, the workflow represents source-group-preserving internal evaluation rather than patient-level or direct external clinical validation.

### 3.2. Dataset, Source Provenance, and Annotation Classes

This study used ER-IHC image data originating from the Universiti Malaya Medical Centre resource [36]. The source literature contains two related dataset descriptions that should be distinguished. Earlier work on whole-slide ER assessment reports a 37-WSI subset used for ER-IHC scoring and associated region-level analysis, whereas the subsequently expanded and revalidated image resource describes 44 ER-IHC whole-slide images acquired using a 3DHistech Panoramic DESK scanner at 20× magnification [37]. In that expanded resource, five regions of interest (ROIs) were extracted from each of the 44 slides, yielding 220 ROIs in total. The present segmentation experiments use the 220-patch resource and should therefore not be interpreted as a 220-patch expansion of the earlier 37-WSI scoring subset.

The experimental package contained 220 paired RGB images and segmentation masks, each standardized to 512×512 pixels. During provenance auditing, every cleaned image was matched to an image in the original distributed ROI archive. The original filenames contained 44 recurring source identifiers, each associated with exactly five patches. This recovered 44×5 structure is consistent with the published construction of the expanded dataset [37]. These identifiers were therefore used as *recovered-source groups* for the source-group-preserving evaluation described in Section 3.4.

The term “source group” is used deliberately. Although the recovered five-patch grouping is consistent with the published 44-slide, five-ROI-per-slide construction, the distributed patch package does not provide an authoritative mapping that independently verifies each recovered identifier as a patient, specimen, tissue block, or whole-slide identifier. The grouping was therefore used to prevent patches sharing the same recovered-source identifier from crossing data partitions, without making patient-level or independently verified slide-level claims.

Published documentation of the expanded dataset provides additional annotation provenance. Five ROIs per slide were used to construct a 22,431-nucleus dataset. Nuclei were segmented and initially assigned to four staining categories, after which the segmentation and class assignments were reviewed patch-wise by two collaborating pathologists, with 110 ROIs assigned to each reviewer [37]. During this validation process, segmentation errors and class assignments could be accepted or corrected, including previously unclassified nuclei. The resulting published nucleus counts were 16,209 negative, 1391weak-positive, 3078 moderate-positive, and 1753 strong-positive nuclei.

A mask-semantics audit of the distributed data was performed before the final grouped evaluation. Comparison of the original and cleaned masks, together with the published four-category nucleus distribution, established the foreground label interpretation used throughout this study: C1 corresponds to ER-negative nuclei, C2 to weak-positive nuclei, C3 to moderate-positive nuclei, and C4 to strong-positive nuclei. Label 0 denotes background/non-target pixels and is distinct from the C1 ER-negative foreground category. The numeric label IDs were retained in the computational pipeline to preserve compatibility with the existing masks and trained models.

These five segmentation labels are treated as spatial reference annotations for the present methodological task. Although C1–C4 preserve an ordered staining-intensity structure, the resulting pixel-level segmentation maps are not themselves validated Allred scores, H-scores, percentages of positive tumor nuclei, or slide-level ER classifications. No patient-level treatment or diagnostic endpoint is derived from the segmentation labels in this study.

**Figure 2:**
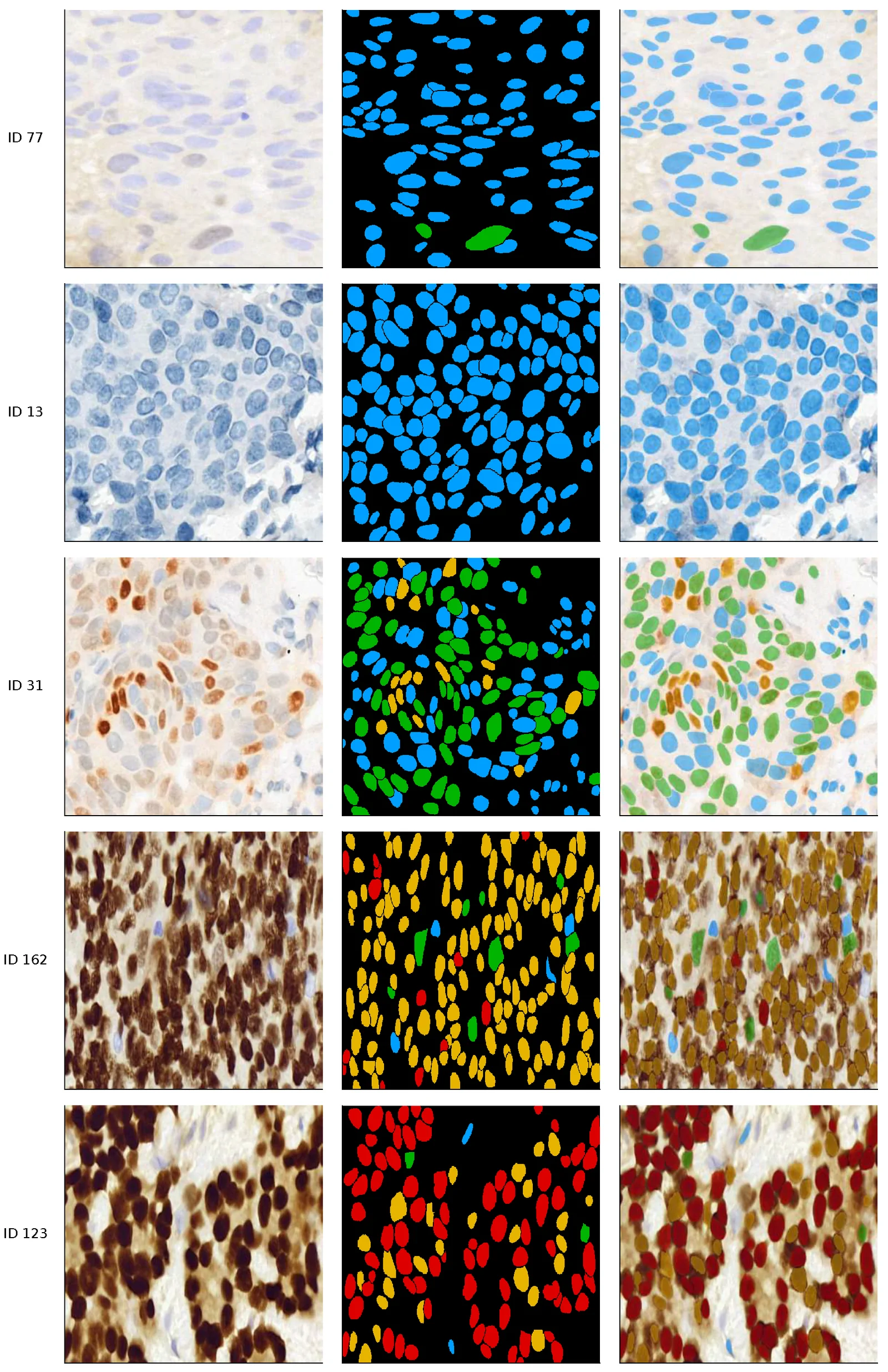
Representative ER-IHC patches, reference masks, and overlays. The five segmentation labels are background/non-target, C1 (ER-negative), C2 (weak-positive), C3 (moderate-positive), and C4 (strong-positive). Background/non-target is distinct from the C1 ER-negative foreground category.

Figure 2 shows representative ER-IHC patches, reference masks, and overlays using the corrected class semantics.

Table 1 summarizes the pixel distribution of the cleaned five-class masks. Background/non-target accounted for 76.42% of all pixels and C1 ER-negative for 17.70%, whereas the positive-intensity classes C2, C3, and C4 accounted for 1.54%, 2.60%, and 1.75%, respectively. Thus, the principal imbalance within the labelled foreground was between the dominant C1 ER-negative class and the less-prevalent C2–C4 positive-intensity classes.

### 3.3. Preprocessing and Quality Control

Image–mask correspondence was audited before model training. The quality-control procedure confirmed 220 matched image–mask pairs, with no unmatched, unreadable, or corrupted image or mask files. All input images were represented as RGB 512×512 patches.

The segmentation masks were converted to integer label maps with five semantic values: label 0 for background/non-target and labels 1–4 for C1–C4, respectively. In the cleaned masks and manuscript visualizations, the foreground classes were represented as cyan for C1, green for C2, yellow for C3, and red for C4. The original distributed masks also contained pure-blue pixels that were not retained as an additional semantic class in the cleaned modelling masks; these pixels were incorporated into label 0 during the existing mask preparation. When a mask contained an alpha channel, the alpha channel was removed after confirming that doing so did not alter the resulting semantic labels. After conversion, every modelling-mask pixel belonged to one of the five expected integer classes.

Image intensities were scaled to the interval [0,1] and then normalized channel-wise using the ImageNet mean

μ=(0.485,0.456,0.406)

and standard deviation

σ=(0.229,0.224,0.225).

The normalized arrays were transposed to channel-first format and stored as 32-bit floating-point tensors. The same deterministic image normalization and mask conversion were applied for inner validation and outer-test inference; random augmentation was restricted to the training pipeline described in Section 3.5.

### 3.4. Source-Group-Preserving Nested Evaluation and Independence Audit

The final evaluation used a source-group-preserving nested five-fold design. The 44 recovered five-patch source groups described in Section 3.2 were treated as indivisible partitioning units. A deterministic grouped assignment (seed 20260810) allocated the source groups to five outer folds containing 9, 9, 9, 9, and 8 groups, corresponding to 45, 45, 45, 45, and 40 patches, respectively. Each recovered-source group was assigned to exactly one outer fold.

For each outer iteration, one grouped fold was reserved as the untouched outer-test partition and the other four folds formed the development set. The four development folds then rotated once each as inner validation. When a 45-patch fold served as inner validation, the corresponding inner-training set contained either 130 or 135 patches depending on whether the 40-patch fold remained in the training partition. When the 40-patch fold served as inner validation, the training set contained 135 patches. Consequently, across the 20 nested splits, inner-training sets contained 130 or 135 patches, inner-validation sets contained 40 or 45 patches, and outer-test sets contained 40 or 45 patches.

The partition audit was performed at both patch and recovered-source levels. In every nested split, the intersection between inner-training, inner-validation, and outer-test patch identifiers was empty. The corresponding recovered-source-group intersections were also empty. Across the complete outer evaluation, every one of the 220 patches and every one of the 44 recovered-source groups appeared in exactly one outer-test fold. Thus, no recovered-source group contributed patches to more than one partition within a nested split.

The outer-test partition was not used for training, AMC updates, checkpoint selection, hyperparameter selection, threshold tuning, or post-test model selection. For each condition and outer fold, four inner models were selected using only their corresponding inner-training–validation partitions and were subsequently carried forward to the frozen outer-test ensemble described in Section 3.1.

The grouped assignment sought to retain representation of the less-prevalent positive-intensity classes while preserving source-group integrity. Because each five-patch source group had to remain intact, exact equality of class composition across outer folds was neither possible nor imposed. Nevertheless, C2, C3, and C4 were represented in every outer fold. The number of C4-containing patches ranged from 11 to 15 across the five outer folds, rather than being artificially equalized. Table 2 reports the resulting grouped outer-fold composition; more detailed pixel-level and class-presence distributions are provided in Supplementary Figures S1–S3.

The five outer folds were treated as paired evaluation blocks for the primary condition-level statistical comparisons. Pixel-level and pooled patch-level summaries were not treated as independent inferential observations. Source-group-resampling analyses were used separately as supportive dependence-aware sensitivity analyses, as specified in Section 3.16. The recovered-source groups reduce cross-partition dependence relative to the previous patch-only split, but, as noted in Section 3.2, they are not interpreted as independently verified patient or whole-slide identifiers.

### 3.5. Training-Only Data Augmentation

Data augmentation was applied only to inner-training samples. The augmentation policy combined conservative geometric transformations with mild appearance perturbations intended to increase local variability without altering the semantic interpretation of the ER-IHC labels.

Horizontal and vertical flips were each applied independently with probability 0.50. Random rotation by a multiple of 90° was applied with probability 0.50. Mild affine augmentation was applied with probability 0.45 and comprised rotation within −12° to 12°, isotropic scaling between 0.92 and 1.08, and horizontal and vertical translations of up to 4% of the image dimensions.

Appearance augmentation included mild brightness and contrast perturbation, gamma adjustment, small HSV perturbations, Gaussian blur, and low-amplitude Gaussian noise. Geometric transformations were applied synchronously to each image and its corresponding segmentation mask, whereas appearance transformations were applied to the image only.

Augmentation was disabled for inner-validation and outer-test data. These partitions were evaluated using the complete 512×512 patches with the deterministic preprocessing described in Section 3.3. No random crop selection, geometric augmentation, or appearance augmentation was applied during validation or outer-test inference.

### 3.6. Comparative Models and Experimental Roles

The primary source-group-preserving nested comparison was designed to isolate the effects of imbalance-handling strategies rather than architectural differences. All six conditions therefore used the same ResUNet-DS backbone, training duration, augmentation policy, checkpoint-selection criterion, and outer-test ensembling procedure. The controlled factors were class weighting, crop-selection strategy, and inclusion or exclusion of the Focal–Tversky objective component.

The six conditions comprised unweighted random training, weighted random training, weighted fixed-minority sampling, weighted random training with Focal–Tversky, weighted adaptive minority curriculum (AMC), and weighted adaptive AMC with Focal–Tversky. AMC-Focal was not treated as a distinct network architecture: AMC modified only the training-crop selection process, whereas Focal–Tversky modified the optimization objective. The individual-model inference architecture was identical across all six conditions.

Additional architecture and training-strategy experiments had been conducted during model development. These included U-Net, Attention U-Net, a TransUNet-style model, nnU-Net, DeepLabV3-ResNet50, ResUNet-DS, component ablations, Focal–Tversky parameter-sensitivity experiments, ordinal-loss variants, and C4-protective sampling. These experiments used the earlier non-nested five-fold training–validation protocol and are therefore reported only as supporting development-stage analyses. They were not included in the primary source-group-preserving outer-test comparison, were not used for the confirmatory fold-level statistical comparisons, and did not influence the final condition-level conclusions. The TransUNet-style comparator was implemented as a lightweight convolutional encoder–decoder with a transformer bottleneck and was not intended as a reproduction of the complete official TransUNet configuration. The custom PyTorch development comparators were implemented using PyTorch version 2.1.2 with CUDA 12.1 support and were trained for 120 epochs using 320×320 training crops and complete 512×512 validation patches, whereas nnU-Net retained its framework-specific preprocessing, optimization, and checkpoint-management procedures.

### 3.7. ResUNet-DS Backbone and Training-Only Imbalance Components

All six primary conditions used the same five-class ResUNet-DS backbone. The network accepted three-channel RGB input and used 32 base channels, residual encoder–decoder blocks, skip connections, spatial-and-channel squeeze-and-excitation recalibration (scSE), and auxiliary deep-supervision outputs. The individual model contained approximately 8.17 million trainable parameters.

The architecture produced predictions for five labels: background/non-target and foreground classes C1–C4. The latter corresponded to C1 ER-negative, C2 weak-positive, C3 moderate-positive, and C4 strong-positive, as established in Section 3.2.

AMC and Focal–Tversky were training-only components layered around this common backbone. AMC altered which 320×320 crops were presented during optimization, whereas Focal–Tversky altered the loss function. Neither component changed the feature-extraction pathway, decoder structure, number of output channels, trainable parameter count, or individual-model inference graph. Outer-test predictions for every condition were subsequently obtained from a four-model probability ensemble, as described in Section 3.4 and detailed further in the checkpoint-and-ensembling subsection below.

Foreground-ordinal Earth Mover’s Distance was examined only in an earlier development-stage ablation and was not included in any of the six primary nested training conditions.

**Figure 3:**
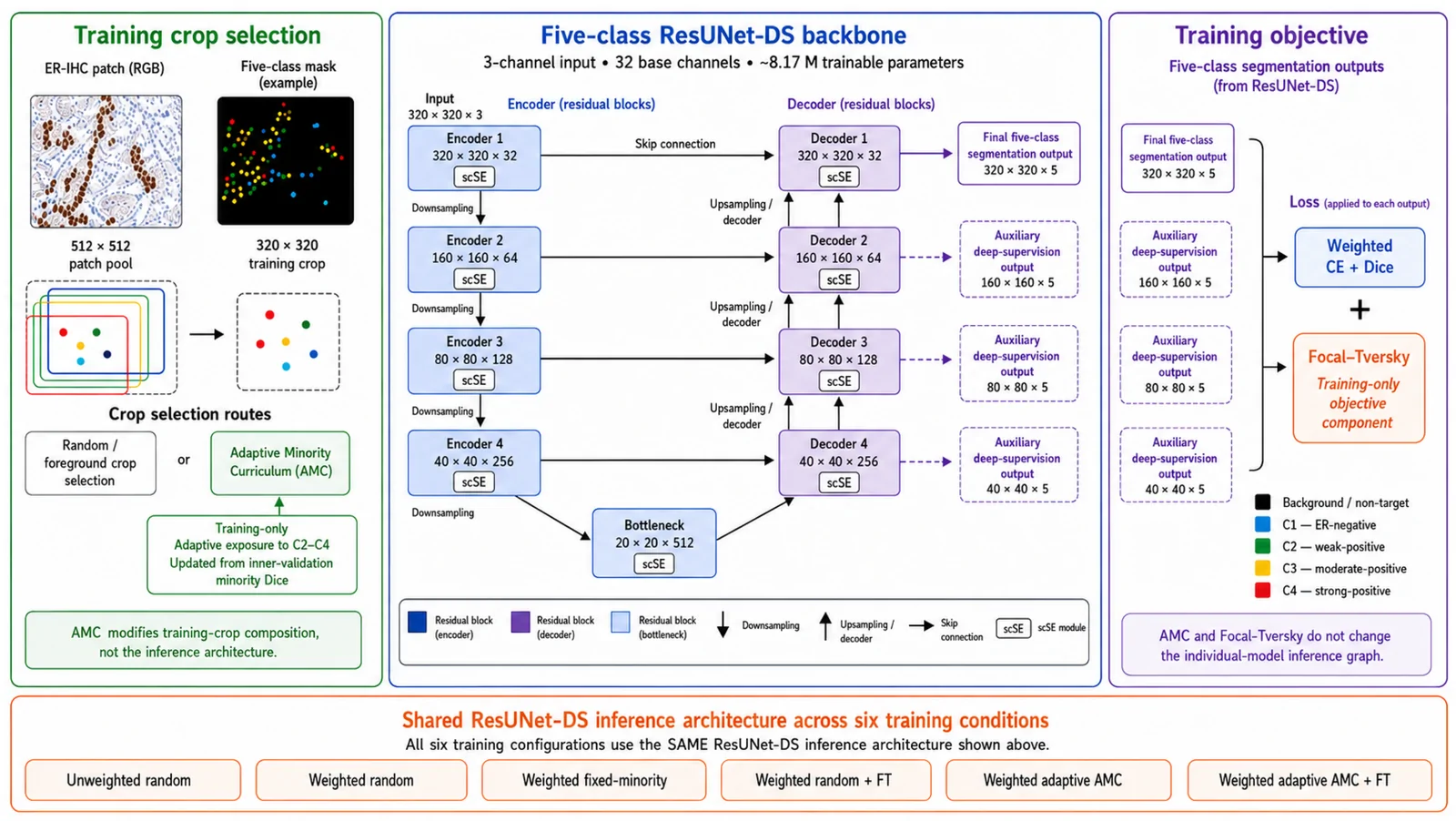
Common five-class ResUNet-DS backbone and training-only imbalance-handling components. All six primary conditions used the same residual encoder–decoder inference architecture with scSE recalibration, skip connections, and deep supervision. Adaptive minority curriculum (AMC) modified training-crop composition, while Focal–Tversky modified the training objective. Neither component altered the individual-model inference graph.

Figure 3 distinguishes the common inference architecture from the AMC crop-selection mechanism and the Focal–Tversky training objective.

### 3.8. Adaptive Minority Curriculum Sampling

AMC was used only in the two adaptive-sampling conditions and operated entirely within each inner-training–validation split. Its purpose was to adapt the frequency with which training crops were centered on the less-prevalent positive-intensity classes C2–C4. No AMC update used outer-test information.

At training epoch *t*, crop selection was governed by a curriculum probability $p_{t}$. When the minority branch was selected, one of C2, C3, or C4 was sampled according to class probabilities computed from the pixel counts of the current inner-training partition:

(1) $$q_{c}=\frac{1/\sqrt{N_{c}+1}}{\sum_{k\in \{2,3,4\}}1/\sqrt{N_{k}+1}},\;c\in \left\{2,3,4\right\},$$

where $N_{c}$ denotes the number of inner-training pixels belonging to class *c*. The inverse-square-root weighting increased the sampling probability of rarer positive-intensity classes while avoiding an exclusive focus on the rarest class.

For each training iteration, a random value r∼U(0,1) determined the crop-selection branch. If $r<p_{t}$, a class c∈{C2,C3,C4} was drawn according to $q_{c}$, and the crop was centered on a pixel belonging to that class. If no valid center for the selected class was available in the sampled mask, the procedure fell back to any C1–C4 foreground pixel. The next probability interval, up to $p_{t}+0.20$, used foreground-centered sampling; remaining draws used random cropping. If a valid centered crop could not be obtained, random cropping was used as the final fallback.

The curriculum probability was initialized as

$$p_{0}=0.25$$

and updated after every training epoch using the C2–C4 mean Dice measured on the corresponding inner-validation partition:

(2) $$p_{t+1}=\mathrm{clip}\left[p_{t}+g\left(D_{\mathrm{target}}-D_{\mathrm{minority},t}\right),p_{\min},p_{\max}\right],$$

where $D_{\mathrm{minority},t}$ is the mean validation Dice over C2–C4 after epoch *t*. The fixed curriculum parameters were

$$D_{\mathrm{target}}=0.70,\;g=0.20,\;p_{\min}=0.20,\;p_{\max}=0.85.$$

The clipping operator was defined as

clip(z,a,b)=min{max{z,a},b}.

Accordingly, $p_{t}$ increased when inner-validation minority Dice was below the target value and decreased when minority Dice exceeded that target, subject to the predefined lower and upper bounds. Each AMC trajectory was therefore determined exclusively by the data available within its corresponding inner split.

Algorithm 1 summarizes the training-time sampling procedure.**Algorithm 1** Adaptive Minority Curriculum Sampling**Require:** Inner-training image–mask pair (x,y)**Require:** Crop size 320×320**Require:** Minority classes {C2,C3,C4}**Require:** Curriculum probability $p_{t}$**Require:** Foreground-branch interval 0.20**Require:** Minority-class probabilities $q_{c}$  1:**for** each inner-training iteration **do**  2:    Load (x,y) from the inner-training partition  3:    Draw r∼U(0,1)  4:    **if** $r<p_{t}$ **then**  5:        Select c∈{C2,C3,C4} according to $q_{c}$  6:        Select a crop center from pixels belonging to class *c*  7:        **if** no valid class-*c* center is available **then**  8:           Select a center from any C1–C4 foreground pixel  9:        **end if**10:    **else if** $r<p_{t}+0.20$ **then**11:        Select a center from any C1–C4 foreground pixel12:    **else**13:        Select a random crop14:    **end if**15:    **if** no valid centered crop is available **then**16:        Use random cropping17:    **end if**18:    Extract a 320×320 crop19:    Apply the training-only augmentation policy20:**end** **for**21:Compute inner-validation $D_{\mathrm{minority},t}$22:Update $p_{t+1}$ using Equation (2)

### 3.9. Training Objectives

All six primary conditions used a common segmentation objective consisting of cross-entropy and foreground soft Dice loss. Class weighting was enabled only in the weighted conditions, and Focal–Tversky loss was added only in the two conditions for which it was explicitly specified. Deep supervision was applied consistently across all conditions.

For the main network output, the objective at epoch *t* was

(3) $$L_{\mathrm{main}}\left(t\right)=L_{\mathrm{ce}}+L_{\mathrm{dice}}+\lambda_{\mathrm{ft}}\left(t\right)\;L_{\mathrm{ft}},$$

where $\lambda_{\mathrm{ft}}\left(t\right)=0$ for conditions that did not use Focal–Tversky optimization. With *J* auxiliary deep-supervision outputs, the complete training objective was

(4) $$L_{\mathrm{total}}\left(t\right)=L_{\mathrm{main}}\left(t\right)+\lambda_{\mathrm{aux}}\frac{1}{J}\sum_{j=1}^{J}L_{\mathrm{aux}}^{\left(j\right)},\;\lambda_{\mathrm{aux}}=0.10.$$

Each auxiliary output used the same condition-specific cross-entropy formulation and foreground soft Dice loss as the main output, but no Focal–Tversky term was applied to the auxiliary losses.

#### 3.9.1. Cross-Entropy Weighting

For weighted conditions, class weights were computed independently within each inner-training partition so that no validation or outer-test label distribution contributed to the weighting scheme. Let $f_{c}$ denote the relative pixel frequency of class *c* in the corresponding inner-training partition. The unnormalized class weight was

(5) $${\tilde{w}}_{c}=\frac{1}{\sqrt{f_{c}+\epsilon_{w}}},\;\epsilon_{w}=10^{-8}.$$

The five weights were then normalized by their mean and clipped to limit extreme weighting:

(6) $$w_{c}=\mathrm{clip}\left(\frac{{\tilde{w}}_{c}}{\frac{1}{5}\sum_{k=0}^{4}{\tilde{w}}_{k}},0.25,4.0\right),\;c\in \left\{0,\ldots ,4\right\}.$$

For the unweighted condition, $w_{c}=1$ for all five classes. Cross-entropy was evaluated over background/non-target and C1–C4 using the weights defined for the corresponding condition.

#### 3.9.2. Foreground Soft Dice Loss

Soft Dice loss was calculated over the four foreground classes and excluded background/non-target. For foreground class *c*,

(7) $${\mathrm{Dice}}_{c}^{\mathrm{soft}}=\frac{2\sum_{i}p_{i,c}y_{i,c}+\epsilon }{\sum_{i}p_{i,c}+\sum_{i}y_{i,c}+\epsilon },\;c\in \left\{1,2,3,4\right\},$$

where $p_{i,c}$ is the predicted probability for class *c* at pixel *i*, $y_{i,c}$ is the corresponding one-hot reference indicator, and ϵ is a numerical-stability constant. The foreground soft Dice loss was

(8) $$L_{\mathrm{dice}}=\frac{1}{4}\sum_{c=1}^{4}\left(1-{\mathrm{Dice}}_{c}^{\mathrm{soft}}\right).$$

#### 3.9.3. Focal–Tversky Loss

Focal–Tversky loss was used only in the two designated Focal–Tversky conditions. For each foreground class *c*, the implemented Tversky index was

(9) $${\mathrm{TI}}_{c}=\frac{{\mathrm{TP}}_{c}+\epsilon }{{\mathrm{TP}}_{c}+\alpha {\mathrm{FP}}_{c}+\beta {\mathrm{FN}}_{c}+\epsilon },$$

with

(10) $${\mathrm{TP}}_{c}=\sum_{i}p_{i,c}y_{i,c},$$

(11) $${\mathrm{FP}}_{c}=\sum_{i}p_{i,c}\left(1-y_{i,c}\right),$$

(12) $${\mathrm{FN}}_{c}=\sum_{i}\left(1-p_{i,c}\right)y_{i,c}.$$

The foreground-averaged Focal–Tversky loss was

(13) $$L_{\mathrm{ft}}=\frac{1}{4}\sum_{c=1}^{4}{\left(1-{\mathrm{TI}}_{c}\right)}^{\gamma }.$$

The fixed parameters were

α=0.70,β=0.30,γ=2.0.

Under the implemented convention, α multiplies the false-positive term and β multiplies the false-negative term; the selected configuration therefore imposed the larger Tversky penalty on false-positive foreground predictions. These coefficients were treated as fixed empirical training settings rather than as theoretically optimal values.

To avoid introducing the additional loss term at full strength from the beginning of optimization, its contribution was scheduled over training. Specifically,

(14) $$\lambda_{\mathrm{ft}}\left(t\right)=\left\{\begin{array}{cc} 0,\; & t<5,\\ 0.50\;\frac{t-5}{10},\; & 5\leq t<15,\\ 0.50,\; & t\geq 15. \end{array}\right.$$

Thus, the Focal–Tversky contribution was absent during the initial five epochs, increased linearly over the next ten epochs, and remained at 0.50 thereafter.

#### 3.9.4. Development-Stage Ordinal Objective

A foreground-ordinal Earth Mover’s Distance term was examined during earlier development-stage ablation experiments to assess whether explicitly incorporating the C1–C4 ordering into the optimization objective provided additional benefit. That term was not retained in the final six-condition comparison and therefore did not contribute to any model evaluated in the primary source-group-preserving analysis. Its formulation and development-stage results are reported in the Supplementary Materials.

### 3.10. Nested Training Configuration

Each inner model was trained for 120 epochs using AdamW with an initial learning rate of $3\times 10^{-4}$, weight decay of $1\times 10^{-4}$, and a training batch size of 2. Training was performed on 320×320 crops, whereas inner validation used complete 512×512 patches with batch size 1. No random augmentation or crop selection was applied during inner validation.

Automatic mixed precision was enabled when CUDA was available. Gradients were clipped to a maximum norm of 1.0 before each optimizer update, and two data-loading workers were used.

A base random seed of 42 was used. To obtain a deterministic, split-specific seed for each nested run, the seed for outer fold *o* and inner-validation fold *v* was defined as

(15) $$s_{o,v}=42+100o+v.$$

The same training duration, optimizer settings, crop size, augmentation policy, gradient-clipping rule, and checkpoint-selection procedure were applied across all six primary conditions. Differences between conditions were restricted to the class-weighting, crop-selection, and Focal–Tversky components defined in the preceding subsections. The complete common training configuration used for the six primary conditions is summarized in Table 3.

### 3.11. Primary Experimental Conditions

The primary comparison comprised six training conditions that differed only in class weighting, crop-selection strategy, and use of the Focal–Tversky objective. All other training and evaluation settings were held constant as described in the preceding subsections. Table 4 summarizes the controlled configuration of each condition.

Development-stage architecture comparisons, component ablations, parameter-sensitivity analyses, ordinal-loss variants, and alternative sampling experiments are reported separately in the Supplementary Materials and were not part of this primary six-condition comparison.

### 3.12. Inner Checkpoint Selection and Outer-Test Ensembling

Checkpoint selection was performed independently within each inner-training–validation split. After every training epoch, model performance on the corresponding inner-validation partition was summarized using the fixed composite score

(16) $$S_{\mathrm{inner}}=0.60D_{\mathrm{minority}}+0.30D_{\mathrm{foreground}}+0.10\kappa_{\mathrm{fg}},$$

where $D_{\mathrm{minority}}$ denotes the mean Dice over the positive-intensity classes C2–C4, $D_{\mathrm{foreground}}$ denotes the mean Dice over all foreground classes C1–C4, and $\kappa_{\mathrm{fg}}$ denotes foreground-only weighted kappa. The checkpoint achieving the highest $S_{\mathrm{inner}}$ was retained for that inner split. The same selection criterion was applied to all six experimental conditions.

For each condition and outer fold, the four checkpoints selected from the corresponding inner splits were used to form the outer-test ensemble. Each model processed the complete 512×512 outer-test patch and produced five-class logits from the main output head. SoftMax was applied to each model independently, yielding per-pixel class probabilities $p_{i,c}^{\left(m\right)}$ for model *m*, pixel *i*, and class *c*. Ensemble probabilities were obtained by arithmetic averaging:

(17) $${\bar{p}}_{i,c}=\frac{1}{4}\sum_{m=1}^{4}p_{i,c}^{\left(m\right)}.$$

The final segmentation label at pixel *i* was then assigned as

(18) $${\hat{y}}_{i}=\underset{c\in \{0,\ldots ,4\}}{\arg\max}\;{\bar{p}}_{i,c}.$$

Only the main segmentation output was used for outer-test inference; auxiliary deep-supervision outputs were training-only. Outer-test patches were evaluated at their full spatial resolution without evaluation-time cropping or augmentation. No threshold tuning, probability recalibration, checkpoint replacement, model exclusion, post-processing selection, or other model-selection decision was made using outer-test performance.

### 3.13. Segmentation and Ordinal Evaluation Metrics

Performance was assessed using complementary segmentation-overlap and ordinal-agreement measures. Segmentation metrics comprised class-specific Dice scores for C1–C4, mean foreground Dice, mean foreground intersection over union (IoU), mean Dice over the less-prevalent positive-intensity classes C2–C4, and foreground macro precision and macro recall. Mean foreground metrics were calculated over C1–C4, whereas the C2–C4 Dice summary specifically characterized performance on the weak-, moderate-, and strong-positive-intensity classes.

For class *c*, Dice was defined as

(19) $${\mathrm{Dice}}_{c}=\frac{2{\mathrm{TP}}_{c}}{2{\mathrm{TP}}_{c}+{\mathrm{FP}}_{c}+{\mathrm{FN}}_{c}},$$

and IoU as

(20) $${\mathrm{IoU}}_{c}=\frac{{\mathrm{TP}}_{c}}{{\mathrm{TP}}_{c}+{\mathrm{FP}}_{c}+{\mathrm{FN}}_{c}}.$$

Mean foreground Dice and mean foreground IoU were obtained by averaging their respective class-level values over C1–C4. The C2–C4 Dice summary was defined as

(21) $$D_{C2-C4}=\frac{1}{3}\sum_{c=2}^{4}{\mathrm{Dice}}_{c}.$$

Because C1–C4 form an ordered foreground sequence from ER-negative to increasing positive staining intensity, overlap-based evaluation was supplemented by foreground-only quadratically weighted kappa and foreground-ordinal mean absolute error (MAE). These ordinal metrics were calculated from the C1–C4 submatrix of the confusion matrix and therefore included only pixels for which both the reference and predicted labels belonged to the foreground classes.

For weighted kappa, disagreement between true-foreground class *a* and predicted-foreground class *b* was assigned the quadratic weight

(22) $$w_{ab}={\left(\frac{\left|a-b\right|}{3}\right)}^{2},\;a,b\in \left\{1,2,3,4\right\}.$$

Let $O_{ab}$ denote the observed normalized foreground confusion matrix and $E_{ab}$ the corresponding matrix expected from the observed row and column marginals. Foreground weighted kappa was then

(23) $$\kappa_{\mathrm{fg}}=1-\frac{\sum_{a=1}^{4}\sum_{b=1}^{4}w_{ab}O_{ab}}{\sum_{a=1}^{4}\sum_{b=1}^{4}w_{ab}E_{ab}}.$$

Thus, disagreement between more widely separated intensity classes received a progressively larger penalty.

Foreground-ordinal MAE was defined as

(24) $${\mathrm{MAE}}_{\mathrm{ord}}=\frac{1}{N_{\mathrm{fg}\to \mathrm{fg}}}\sum_{i=1}^{N_{\mathrm{fg}\to \mathrm{fg}}}\left|{\hat{y}}_{i}-y_{i}\right|,$$

where $y_{i},{\hat{y}}_{i}\in \left\{1,2,3,4\right\}$ and $N_{\mathrm{fg}\to \mathrm{fg}}$ denotes the number of pixels for which both reference and prediction were foreground. Lower ordinal MAE therefore indicates that foreground misclassifications occurred over shorter distances in the ordered C1–C4 sequence.

Exact foreground agreement and adjacent and distant ordinal-error rates were retained as descriptive supporting measures. An adjacent error had ordinal distance $\left|{\hat{y}}_{i}-y_{i}\right|=1$, whereas a distant error had ordinal distance of at least two. Foreground-to-background and background-to-foreground errors were excluded from the ordinal metrics but remained represented by the segmentation-overlap, prevalence, and spatial-error analyses.

### 3.14. Model-Derived Descriptive Outputs

The outer-test ensemble predictions were also used to derive descriptive spatial and probabilistic outputs beyond the primary segmentation and ordinal metrics. These included C1–C4 segmentation maps, class-specific probability maps, predicted class proportions, predictive-entropy maps, four-model disagreement maps, and ordinal-error maps. These quantities were calculated from the predefined outer-test predictions and were used for descriptive analysis and qualitative inspection only.

Predicted class proportions summarized the pixel area assigned to the individual C1–C4 categories and to the combined positive-intensity classes C2–C4. These quantities represent model-derived pixel-area summaries rather than counts or proportions of individual tumor nuclei. Accordingly, they were not interpreted as percentage ER positivity, Allred score, H-score, slide-level ER status, or any other clinical biomarker score.

Predictive entropy was derived from the ensemble-averaged class probabilities and described the dispersion of the predictive distribution at each pixel. Ensemble disagreement was assessed separately from variation among the four constituent model predictions. These two quantities were retained as distinct descriptions of predictive behavior and were not treated as interchangeable measures of calibrated uncertainty.

Ordinal-error maps provided a spatial representation of correct foreground classification, ordinal under-estimation and over-estimation, foreground missed as background/non-target, and background/non-target predicted as foreground. Probability, entropy, disagreement, and error maps were used to characterize model behavior rather than as validated clinical confidence estimates, independent diagnostic outputs, or automated error-screening tools.

### 3.15. Computational Complexity and Deployment Scope

AMC and Focal–Tversky were training-only components and therefore did not alter the individual ResUNet-DS inference architecture, parameter count, or operation count. A single ResUNet-DS model contained approximately 8.17 million trainable parameters.

Computational profiling performed during model development evaluated single-model FP32 inference on an NVIDIA RTX A5000 GPU. The detailed profiling protocol, operation-counting assumptions, latency, throughput, memory consumption, and architecture-level comparisons are reported in the Supplementary Materials. These measurements are retained as supporting implementation characteristics rather than as performance claims for the final ensemble evaluation.

For each condition and outer fold, final predictions were obtained by averaging the probability maps from four independently selected inner models. The resulting ensemble therefore required four model forward passes per patch, followed by probability averaging and pixel-wise argmax prediction. The end-to-end latency, memory requirement, energy consumption, and throughput of this four-model ensemble were not measured. Consequently, the earlier single-model profiling should not be interpreted as the computational cost of deploying the complete outer-test ensemble.

### 3.16. Fold-Level Statistical Analysis

The five source-group-preserving outer folds were treated as paired evaluation blocks for condition-level comparison. Individual pixels, patches, and recovered-source groups were not used to inflate the sample size of the fold-level statistical tests. Condition-level performance was summarized as mean ± standard deviation across the five outer folds, while pooled pixel- and patch-level quantities were treated as descriptive summaries.

The unweighted-random condition was used as the reference for the primary comparative analysis. Each of the five weighted or minority-sensitive conditions was compared with this reference for five predefined endpoints: mean foreground Dice, mean C2–C4 Dice, mean foreground IoU, foreground quadratically weighted kappa, and foreground-ordinal MAE. This produced 25 paired comparisons in total, with n=5 paired outer-fold observations for every comparison.

For each comparator and endpoint, the fold-level difference was defined as

(25) $$d_{o}=M_{o,\mathrm{cmp}}-M_{o,\mathrm{ref}},\;o=1,\ldots ,5,$$

where $M_{o,\mathrm{cmp}}$ and $M_{o,\mathrm{ref}}$ denote the metric values for the comparator and unweighted reference, respectively, on outer fold *o*. The mean paired difference and its 95% *t*-interval across the five-fold differences were reported as descriptive effect estimates.

Standardized paired effect size was expressed using Cohen’s $d_{z}$,

(26) $$d_{z}=\frac{\bar{d}}{s_{d}},$$

where $\bar{d}$ is the mean paired difference and $s_{d}$ is the sample standard deviation of the five paired differences. Rank-biserial effect size and the numbers of fold-level comparator wins, losses, and ties were also reported to describe the direction and consistency of the paired differences.

Exact two-sided Wilcoxon signed-rank tests were used for the paired outer-fold comparisons. With five paired fold observations, the exact test has coarse resolution; when all five paired differences are non-zero, the smallest attainable two-sided *p*-value is 0.0625. The resulting *p*-values were therefore interpreted together with the paired effect estimates and fold-wise consistency rather than as stand-alone evidence.

Multiplicity correction was applied separately within each endpoint. For a given endpoint, the five comparator-versus-reference *p*-values were adjusted using the Holm procedure. Thus, the multiplicity-controlled analysis comprised five families of five comparisons rather than all pairwise contrasts among the six conditions. Statistical significance was assessed at α=0.05 after Holm adjustment.

The outer folds are cross-validation evaluation blocks rather than independent biological units. Accordingly, the fold-level tests and their 95% intervals quantify consistency of the observed condition differences across the five grouped outer evaluations and should not be interpreted as patient-level or whole-slide-level inferential evidence.

#### Source-Group-Resampling Sensitivity Analysis

As a dependence-aware sensitivity analysis, uncertainty intervals were also estimated by paired source-group resampling. The resampling unit was the recovered five-patch source group rather than the individual patch or pixel. Source groups were resampled with replacement within each of the five fixed outer folds, and the same bootstrap draw was applied across all six model conditions so that condition comparisons remained paired.

A total of 20,000 stratified bootstrap replicates were generated using a fixed random seed of 20260812. For each condition, pooled source-group-resampled estimates and 95% bootstrap intervals were calculated. Paired comparator-versus-reference differences were recomputed using the same bootstrap draws for the five primary segmentation and ordinal endpoints.

These intervals were used as supportive internal sensitivity analyses to assess whether conclusions were dependent on particular recovered-source groups. They were not used as additional multiplicity-controlled significance tests and were not interpreted as patient-level, specimen-level, or whole-slide-level confidence intervals because the recovered-source groups do not constitute independently verified biological identifiers.

### 3.17. Post Hoc Probability Quality, Prevalence, and Error-Ranking Analysis

All secondary probability analyses were performed using the frozen outer-test outputs of the six primary conditions after completion of model training and checkpoint selection. The saved ensemble probabilities, final argmax predictions, and four constituent inner-model predictions were analyzed without modifying the trained models or their outer-test outputs. No temperature scaling, probability recalibration, threshold tuning, calibration-based refitting, checkpoint replacement, model exclusion, or post-test model selection was performed.

Probability quality, predicted foreground composition, predictive entropy, ensemble disagreement, and error-ranking performance were treated as supporting analyses distinct from the primary segmentation and ordinal endpoints. Individual pixels and patches were not treated as independent inferential units. Where uncertainty intervals were reported, the paired source-group resampling procedure described in the source-group-resampling sensitivity analysis was used.

#### 3.17.1. Probability Quality Across Evaluation Scopes

Probability quality was evaluated at four complementary scopes. Let $p_{i,c}$ denote the ensemble-averaged probability assigned to class c∈{0,…,4} at pixel *i*.

The first scope evaluated five-class probability quality over all pixels. The second evaluated the same five-class prediction problem after restricting the analysis to pixels whose reference labels were C1–C4. In this true-foreground multiclass analysis, the original five-class probability vector was retained without renormalization; probability assigned to background/non-target therefore remained part of the evaluated prediction.

The third scope evaluated binary foreground presence over all pixels. The foreground probability was

(27) $$q_{i,\mathrm{fg}}=1-p_{i,0},$$

with binary target $1(y_{i}>0)$.

The fourth scope examined the allocation of probability between ER-negative foreground and positive-intensity foreground. For pixels with $y_{i}>0$, the probability assigned to C2–C4 conditional on foreground was

(28) $$q_{i,+}=\frac{p_{i,2}+p_{i,3}+p_{i,4}}{\sum_{k=1}^{4}p_{i,k}+\epsilon },$$

with binary target $1(y_{i}\in \left\{2,3,4\right\})$. This conditional task examined positive-intensity probability allocation within known foreground and was not interpreted as a measure of global five-class calibration.

For the multiclass scopes, negative log-likelihood (NLL) was defined as

(29) $${\mathrm{NLL}}_{\mathrm{multi}}=-\frac{1}{N}\sum_{i=1}^{N}\log\left(p_{i,y_{i}}+\epsilon \right),$$

and the multiclass Brier score as

(30) $${\mathrm{Brier}}_{\mathrm{multi}}=\frac{1}{N}\sum_{i=1}^{N}\sum_{c=0}^{4}{\left[p_{i,c}-1\left(y_{i}=c\right)\right]}^{2}.$$

For either binary task with event probability $q_{i}$ and target $z_{i}\in \left\{0,1\right\}$,

(31) $${\mathrm{NLL}}_{\mathrm{bin}}=-\frac{1}{N}\sum_{i=1}^{N}\left[z_{i}\log\left(q_{i}+\epsilon \right)+\left(1-z_{i}\right)\log\left(1-q_{i}+\epsilon \right)\right],$$

and

(32) $${\mathrm{Brier}}_{\mathrm{bin}}=\frac{1}{N}\sum_{i=1}^{N}{\left(q_{i}-z_{i}\right)}^{2}.$$

Expected calibration error was calculated using 15 fixed-width probability bins. For bin sets $B_{b}$,

(33) $${\mathrm{ECE}}_{15}=\sum_{b=1}^{15}\frac{|B_{b}|}{N}\left|\mathrm{obs}\left(B_{b}\right)-\mathrm{conf}\left(B_{b}\right)\right|.$$

For multiclass evaluation, $\mathrm{conf}(B_{b})$ was the mean top-label probability and $\mathrm{obs}(B_{b})$ the corresponding empirical accuracy. For binary evaluation, these quantities were the mean event probability and empirical event frequency, respectively. NLL and Brier score were treated as probability-quality measures, whereas ECE was interpreted as a complementary bin-dependent reliability summary.

The complete frozen predictions were used to calculate NLL, Brier score, and fixed-bin ECE. Pooled estimates, 95% source-group bootstrap intervals, and paired comparator-versus-unweighted differences were obtained using the same 20,000 paired stratified source-group resamples described in the source-group-resampling sensitivity analysis. These intervals were interpreted as supportive sensitivity summaries rather than as additional multiplicity-controlled significance tests.

#### 3.17.2. True-Foreground Class-Specific Calibration

A separate analysis examined probability allocation among the four foreground categories. For pixels with $y_{i}>0$, the C1–C4 probabilities were renormalized as

(34) $$p_{i,c}^{\mathrm{cond}}=\frac{p_{i,c}}{\sum_{k=1}^{4}p_{i,k}+\epsilon },\;c\in \left\{1,2,3,4\right\}.$$

For each of C1–C4, one-versus-rest Brier score and fixed 15-bin ECE were calculated using $p_{i,c}^{\mathrm{cond}}$ and $1(y_{i}=c)$. This analysis characterized relative probability allocation among C1 ER-negative, C2 weak-positive, C3 moderate-positive, and C4 strong-positive after conditioning on reference foreground. It therefore did not evaluate the ability to distinguish foreground from background/non-target.

The paired source-group bootstrap draws were reused to obtain 95% intervals for the class-specific measures and for differences relative to unweighted random training. These class-specific comparisons were exploratory and were not included in the multiplicity-controlled primary segmentation and ordinal endpoint family.

#### 3.17.3. Positive-Intensity Foreground Composition

Preservation of the relative abundance of positive-intensity foreground was evaluated from the final hard ensemble predictions. Let $T_{c}$ denote the number of reference pixels belonging to foreground class *c* and $P_{c}$ the corresponding number assigned to that class by the ensemble argmax prediction. The reference and predicted C2–C4 fractions within foreground were

(35) $$\pi_{+}=\frac{T_{2}+T_{3}+T_{4}}{T_{1}+T_{2}+T_{3}+T_{4}},\;{\hat{\pi }}_{+}=\frac{P_{2}+P_{3}+P_{4}}{P_{1}+P_{2}+P_{3}+P_{4}}.$$

Signed bias and absolute prevalence error were defined as

(36) $$B_{+}={\hat{\pi }}_{+}-\pi_{+},\;E_{+}=\left|{\hat{\pi }}_{+}-\pi_{+}\right|.$$

The principal prevalence-preservation analysis used this combined C2–C4 foreground-composition fraction. Class-specific and overall pixel-prevalence summaries were retained as supporting descriptive quantities.

Uncertainty intervals for prevalence and paired differences in absolute prevalence error were obtained using the same 20,000 paired source-group bootstrap resamples. These quantities describe pixel-area composition only and were not interpreted as percentages of positive tumor nuclei, Allred-score components, H-score components, slide-level ER status, or other clinical biomarker measurements.

#### 3.17.4. Predictive Entropy and Ensemble Disagreement

Predictive entropy quantified dispersion in the ensemble-averaged five-class probability vector. Normalized five-class entropy was

(37) $$U_{i}=-\frac{1}{\log5}\sum_{c=0}^{4}p_{i,c}\log\left(p_{i,c}+\epsilon \right).$$

For true-foreground pixels, conditional entropy was also calculated from the renormalized C1–C4 probabilities in Equation (34):

(38) $$U_{i}^{\mathrm{cond}}=-\frac{1}{\log4}\sum_{c=1}^{4}p_{i,c}^{\mathrm{cond}}\log\left(p_{i,c}^{\mathrm{cond}}+\epsilon \right).$$

Ensemble disagreement was measured independently from the four constituent inner-model hard predictions. For pixel *i*, the variation ratio was

(39) $$V_{i}=1-\frac{\max_{c}\sum_{m=1}^{4}1\left({\hat{y}}_{i}^{\left(m\right)}=c\right)}{4}.$$

Thus, $V_{i}=0$ indicated unanimous hard-label agreement among the four models, whereas larger values represented greater disagreement. Predictive entropy and variation ratio were treated as distinct quantities: entropy describes dispersion of the ensemble-averaged probability vector, whereas variation ratio describes disagreement among the constituent model decisions.

#### 3.17.5. Uncertainty-Based Error Ranking

Error-ranking AUROC quantified the ability of entropy or model disagreement to rank incorrectly classified pixels above correctly classified pixels. Five endpoints were evaluated: all-pixel five-class entropy, all-pixel variation ratio, true-foreground five-class entropy, true-foreground variation ratio, and conditional C1–C4 entropy.

For the true-foreground five-class analysis, reference pixels were restricted to C1–C4, but prediction correctness was assessed using the original five-class prediction; a foreground pixel predicted as background/non-target therefore remained an error. The conditional C1–C4 analysis instead evaluated classification among the four foreground categories after conditioning on reference foreground and renormalizing probabilities over C1–C4.

For reproducible AUROC estimation while limiting the number of pixel observations, deterministic pixel samples were generated once from the reference targets and reused identically across all six conditions. Up to 12,000 pixels per image were sampled without replacement for all-pixel analyses and up to 6000 true-foreground pixels per image for foreground analyses, using a fixed base seed of 20260804.

Pooled AUROC estimates and 95% sensitivity intervals were reconstructed under the same 20,000 paired source-group bootstrap draws using group-level pairwise Mann–Whitney sufficient statistics. The five error-ranking endpoints were also compared across the five paired outer folds using the same reference-versus-comparator framework as the primary condition analysis. Unweighted random training served as the reference, and exact two-sided Wilcoxon signed-rank tests were performed for each of the five comparators. Holm adjustment was applied separately within each error-ranking endpoint across those five comparisons. These analyses were exploratory and remained separate from the multiplicity-controlled primary segmentation and ordinal endpoint family.

Probability-quality measures, calibration summaries, predictive entropy, ensemble disagreement, and error-ranking AUROC describe different properties of model behavior and were not treated as interchangeable measures. In particular, favorable error-ranking AUROC does not establish probability calibration, and neither entropy nor variation ratio was interpreted as a validated clinical confidence score. None of these analyses was used to claim reliable automated error screening, pathologist replacement, or clinical decision support.

### 3.18. External Expression-Ordering and Cross-Marker Stress Analyses

The external-domain experiments were retained as supporting analyses and were not part of the primary source-group-preserving six-condition comparison. Neither external resource contained pixel-level reference masks compatible with the internal five-class C1–C4 annotation scheme. Accordingly, these experiments were not used to estimate external Dice, IoU, ordinal agreement, or other measures of direct five-class ER-IHC segmentation accuracy.

The two analyses addressed different questions. IHC4BC was used to examine whether model-derived ER-IHC spatial summaries preserved ordering with independently available DAB-derived expression variables, whereas HER2-SISH40x was used only as an exploratory cross-marker and cross-stain stress test. Neither analysis was interpreted as direct external validation of the primary segmentation endpoint.

#### 3.18.1. IHC4BC ER-IHC Expression-Ordering Analysis

The IHC4BC resource contains breast cancer immunohistochemistry data for Ki67, ER, PR, and HER2 [6]. The prepared ER-IHC subset comprised 1226 images from seven patients. Images were resized to 512×512 pixels and processed without retraining using a previously selected five-model AMC-Focal ResUNet-DS ensemble from the development-stage experiments. This external-domain ensemble was distinct from the four-model inner ensembles used for each condition and outer fold in the primary source-group-preserving evaluation.

The IHC4BC analysis had been completed during model development and was retained as a supporting expression-ordering analysis. It was not repeated across the six primary source-group-preserving conditions and was not used to select or rank those conditions.

The IHC4BC data did not provide reference C1–C4 segmentation masks. Available image-level DAB-derived variables included mean and median DAB, ER-positive DAB ratio, and weak-, moderate-, and strong-DAB ratios. Model-derived quantities included predicted C1–C4 area ratios, total predicted foreground area, combined C2–C4 area ratio, a normalized model-derived expression summary, foreground-ordinal mean, mean predictive entropy, and mean maximum class probability.

Spearman rank correlation was used as the principal association measure because the analysis concerned preservation of expression-related ordering rather than equality of measurement scales. Pearson correlation was retained as a complementary measure of linear association. Associations were examined at the image level and summarized within patients. Patient-balanced summaries were also examined to reduce the influence of unequal numbers of images contributed by individual patients.

The DAB-derived variables were not treated as pixel-level class annotations, direct surrogates for the internal C1–C4 labels, or clinical reference scores. Associations between model-derived spatial summaries and DAB-derived variables were therefore interpreted only as evidence of expression-ordering consistency. They were not interpreted as external segmentation accuracy, equivalence between internal and external staining categories, agreement with Allred or H-score, patient-level diagnostic performance, or independently validated clinical performance.

#### 3.18.2. HER2-SISH40x Cross-Marker and Cross-Stain Stress Test

A separate exploratory analysis used 387 HER2-SISH40x images to characterize model behavior under substantial cross-marker and cross-stain domain shift. The previously selected development-stage AMC-Focal ResUNet-DS ensemble was applied without retraining.

HER2-SISH40x differs from the internal ER-IHC resource in biomarker, staining process, image appearance, and source-label definition. Accordingly, its labels were not mapped to the internal C1–C4 categories, and no HER2 classification, segmentation, amplification-scoring, or clinical-performance metric was calculated.

Model-derived foreground composition and uncertainty-related outputs were examined descriptively only. The complete protocol and supporting results are reported in the Supplementary Materials. This experiment was interpreted solely as an exploratory out-of-domain stress test and not as evidence of external ER-IHC segmentation validity, HER2 prediction performance, clinical scoring capability, or transferability of the C1–C4 annotation semantics across biomarkers.

## 4. Results

### 4.1. Dataset Imbalance and Outer-Fold Composition

The reference masks exhibited substantial class imbalance at both the pixel and patch levels. As reported in Table 1, background/non-target accounted for 76.42% of all pixels and C1 ER-negative for 17.70%, whereas the positive-intensity classes C2, C3, and C4 accounted for only 1.54%, 2.60%, and 1.75%, respectively. At the patch level, C2 was present in 127 of 220 patches, C3 in 93 patches, and C4 in 65 patches. Thus, the principal foreground imbalance was between the dominant C1 ER-negative category and the substantially less-prevalent C2–C4 positive-intensity categories.

The source-group-preserving outer-fold assignment retained representation of all three positive-intensity classes while keeping each recovered five-patch source group intact. The five outer-test folds contained 9, 9, 9, 9, and 8 source groups, corresponding to 45, 45, 45, 45, and 40 patches, respectively. Exact equality of class composition was not imposed because source-group integrity took precedence over patch-level balancing.

All five outer folds contained reference pixels from C2, C3, and C4. The numbers of C2-containing patches across folds 0–4 were 29, 24, 27, 24, and 23, respectively; the corresponding C3 counts were 21, 15, 22, 17, and 18, and the C4 counts were 15, 12, 14, 13, and 11. C4 therefore remained the most sparsely represented positive-intensity class at patch level, although it was present in every outer-test fold.

The resulting grouped fold composition is reported in Table 2. Supplementary Figures S1–S3 provide additional visual summaries of class-pixel distribution, foreground/positive-intensity balance, and class presence across the five source-group-preserving outer folds.

### 4.2. Primary Nested Segmentation and Ordinal Results

Table 5 summarizes the primary source-group-preserving nested outer-test results for the six controlled training conditions. Values are means and sample standard deviations across the five outer folds. The five columns correspond to the prespecified endpoints used for the paired condition-level comparisons.

Unweighted random training achieved the most favorable numerical mean for all five primary endpoints, with mean foreground Dice of 0.7479, mean C2–C4 Dice of 0.7098, mean foreground IoU of 0.6074, foreground quadratically weighted kappa of 0.9761, and foreground-ordinal MAE of 0.0446. Thus, the additional class-weighting, minority-focused sampling, and Focal–Tversky components did not improve the observed aggregate source-group-preserving outer-fold performance relative to the simpler unweighted reference.

Among the five weighted or minority-sensitive conditions, weighted random training produced the most favorable numerical means across the five primary endpoints. Its mean foreground Dice was 0.7397, mean C2–C4 Dice was 0.7013, and mean foreground IoU was 0.5961, with weighted kappa of 0.9748 and ordinal MAE of 0.0463. Adaptive AMC alone produced slightly lower aggregate overlap and ordinal performance, while combining AMC with Focal–Tversky did not recover the performance of weighted random training.

Class-specific behavior was not identical to the aggregate ordering. For example, weighted random training achieved a slightly higher numerical C2 Dice than the unweighted reference (0.5994 versus 0.5957), despite lower mean C2–C4 Dice and lower overall foreground overlap. This illustrates why individual class improvements were interpreted alongside the aggregate segmentation and ordinal endpoints rather than as evidence of overall condition superiority.

Formal paired outer-fold comparisons, effect sizes, confidence intervals, and multiplicity-adjusted results are reported in the following statistical-results subsection.

### 4.3. Outer-Fold Statistical Comparisons

The unweighted-random condition was compared with each of the five weighted or minority-sensitive conditions across the five source-group-preserving outer folds. The five prespecified endpoints were mean foreground Dice, mean C2–C4 Dice, mean foreground IoU, foreground quadratically weighted kappa, and foreground-ordinal MAE. Table 6 summarizes the paired effect estimates and multiplicity-adjusted results.

For mean foreground Dice, mean C2–C4 Dice, and mean foreground IoU, the unweighted reference outperformed every comparator in all five outer folds. The corresponding rank-biserial effect size was −1.00 for every comparator when differences were defined as comparator minus reference. Mean-foreground-Dice differences ranged from −0.0081 for weighted random training to −0.0158 for weighted fixed-minority sampling. Mean C2–C4 Dice differences ranged from −0.0085 to −0.0191, and mean foreground-IoU differences from −0.0113 to −0.0203.

For foreground-weighted kappa, weighted random, weighted fixed-minority, and weighted adaptive AMC with Focal–Tversky were lower than the unweighted reference in all five folds. Weighted random with Focal–Tversky and weighted adaptive AMC each exceeded the reference in one of five folds, giving rank-biserial effect sizes of −0.867. For ordinal MAE, for which lower values are favorable, the corresponding direction was reversed: the weighted comparators generally produced larger errors than the reference, with weighted random with Focal–Tversky and weighted adaptive AMC showing one favorable fold each.

Despite the consistent numerical direction of many paired differences, none of the 25 exact Wilcoxon comparisons reached p<0.05 before multiplicity correction. Raw exact *p*-values ranged from 0.0625 to 0.125. After Holm adjustment within each endpoint, every adjusted *p*-value was 0.3125. The minimum attainable two-sided exact *p*-value with five non-zero paired differences was 0.0625, reflecting the limited inferential resolution available from five outer-fold evaluation blocks.

The statistical analysis therefore provided no multiplicity-adjusted evidence that any weighted or minority-sensitive condition improved upon the unweighted reference. Instead, the principal finding was directional consistency: unweighted random training achieved the most favorable numerical aggregate performance, particularly for foreground overlap and C2–C4 Dice, while the small number of paired outer folds precluded strong confirmatory inference.

A complementary source-group-resampling analysis was used to assess the sensitivity of these conclusions to the composition of the recovered-source groups. Those results are reported separately below and are interpreted as supportive internal sensitivity evidence rather than patient-level inference.

### 4.4. Results of Source-Group-Resampling Sensitivity Analysis

The complementary source-group-resampling analysis preserved the overall ordering observed across the five outer folds. For unweighted random training, the pooled mean foreground Dice was 0.7494 with a 95% source-group bootstrap interval of [0.7336,0.7603]. The corresponding mean C2–C4 Dice was 0.7116 [0.6905,0.7256], mean foreground IoU was 0.6084 [0.5910,0.6215], foreground quadratically weighted kappa was 0.9764 [0.9683,0.9808], and foreground-ordinal MAE was 0.0443 [0.0289,0.0621].

Across the five comparator conditions, the paired source-group bootstrap differences for all five primary endpoints retained the same directional ordering as the outer-fold analysis. For example, relative to unweighted random training, weighted random training had a mean-foreground-Dice benefit of −0.0090 (95% interval [−0.0150,−0.0027]) and a C2–C4 Dice benefit of −0.0098 ([−0.0174,−0.0017]), where negative benefit indicates poorer performance than the unweighted reference. The corresponding foreground-IoU difference was −0.0122 ([−0.0191,−0.0046]).

The paired source-group bootstrap intervals for the five primary endpoints were below zero for each of the five weighted or minority-sensitive comparators when the effect was expressed so that positive values favored the comparator. Thus, the direction of the aggregate result was not driven by a small number of particular recovered-source groups.

These bootstrap intervals are supportive sensitivity summaries and were not used as additional multiplicity-controlled significance tests. The resampling unit was the recovered five-patch source group, not an independently verified patient or whole-slide identifier. Accordingly, the intervals should not be interpreted as patient-level or whole-slide-level confidence intervals. The formal inferential conclusions remain those of the paired five-outer-fold analysis in Section 4.3.

### 4.5. Post Hoc Probability Quality, Prevalence, and Error-Ranking Results

Probability quality, predicted foreground composition, calibration, predictive entropy, and four-model disagreement were evaluated from the frozen outer-test probability maps without retraining, recalibration, threshold adjustment, or post-test model selection. These secondary analyses were interpreted separately from the primary segmentation and ordinal endpoints. Dependence-aware 95% sensitivity intervals were obtained by resampling the 44 recovered-source groups within their fixed outer folds using the 20,000 paired bootstrap draws described in the source-group-resampling sensitivity analysis.

#### 4.5.1. Probability Quality and Calibration

Probability quality depended strongly on the evaluated pixel scope. Table 7 reports pooled Brier scores for the four predefined probability tasks. Lower values indicate better probability quality.

For five-class probability quality over all pixels, unweighted random training produced the lowest Brier score (0.104940) and negative log-likelihood (0.182772). The same condition was also most favorable for the binary foreground-versus-background task, with Brier score 0.045823 and negative log-likelihood 0.151886. Paired source-group-resampling differences consistently favored the unweighted reference for these global and foreground-detection probability tasks.

The ordering changed after conditioning on pixels known from the reference mask to be foreground. All five weighted or minority-sensitive conditions reduced true-foreground multiclass Brier score relative to the unweighted value of 0.240767. Weighted adaptive AMC produced the lowest numerical Brier score (0.187194), followed closely by weighted random (0.188662) and weighted fixed-minority training (0.190030). For weighted adaptive AMC, the reduction in true-foreground Brier score relative to unweighted training was 0.053573, with a paired source-group bootstrap interval of [0.044919,0.063228]. The corresponding reduction for weighted random training was 0.052105 [0.042725,0.062697].

A similar but smaller conditional effect was observed for the binary C2–C4-versus-C1 task within true foreground. Weighted random produced the lowest Brier score (0.012368) and lowest negative log-likelihood (0.043447), compared with 0.012931 and 0.045812, respectively, for unweighted training. The paired Brier-score benefit for weighted random was 0.000563 [0.000069,0.001153].

Class-specific true-foreground analysis showed that this weighted random advantage was concentrated mainly in C1 and C2. Relative to unweighted training, weighted random reduced one-versus-rest Brier score by 0.000563 [0.000069,0.001153] for C1 ER-negative and by 0.001287 [0.000521,0.002184] for C2 weak-positive. The corresponding intervals for C3 and C4 crossed zero. Fixed 15-bin ECE values were more variable across tasks and conditions, and the paired class-specific ECE intervals crossed zero; ECE was therefore retained as a descriptive, bin-dependent calibration summary rather than as the basis for condition ranking.

**Figure 4:**
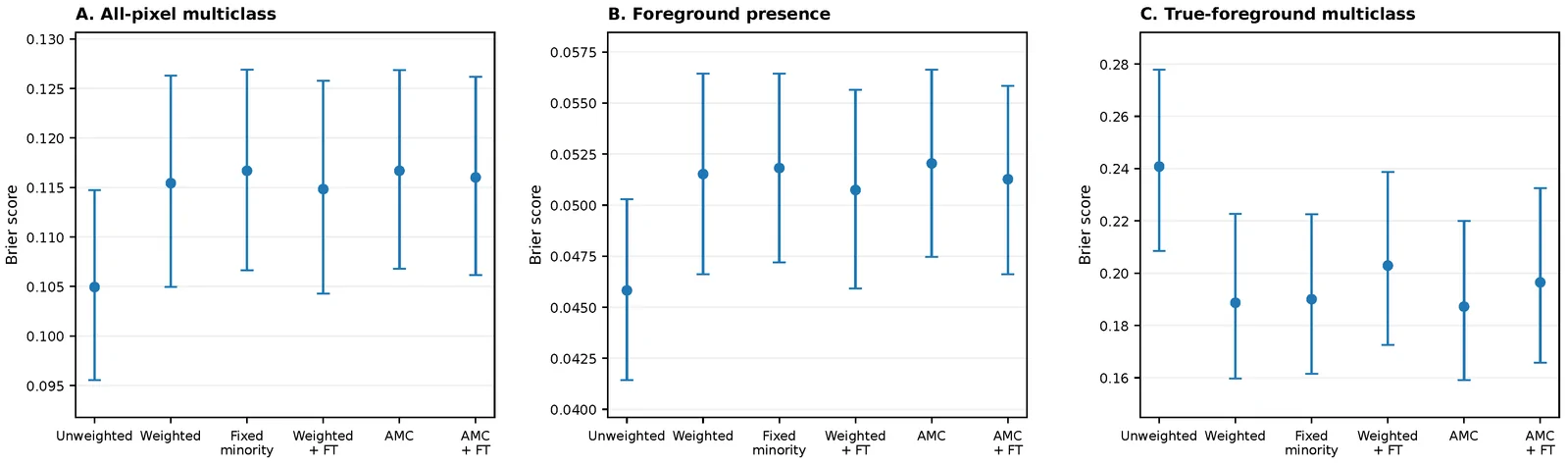
Probability-quality analysis of the six source-group-preserving outer-test conditions. Brier score was evaluated for five-class prediction over all pixels, foreground-versus-background prediction over all pixels, five-class prediction within true foreground, and C2–C4-versus-C1 prediction within true foreground. Lower Brier score indicates better probability quality. Results demonstrate scope-dependent differences between global and conditional foreground probability behavior.

Figure 4 summarizes the principal Brier-score results across the four probability scopes.

#### 4.5.2. Positive-Intensity Composition

The reference C2–C4 fraction within foreground was 0.249306. The corresponding model-derived fractions were 0.242948 for unweighted random, 0.251632 for weighted random, 0.256709 for weighted fixed-minority, 0.257304 for weighted random with Focal–Tversky, 0.255965 for weighted adaptive AMC, and 0.255405 for weighted adaptive AMC with Focal–Tversky.

Weighted random therefore had the smallest numerical absolute bias (0.002326), whereas the unweighted reference had a signed bias of −0.006358. However, the 95% source-group bootstrap intervals for the signed biases included zero for all six conditions. Moreover, all paired bootstrap intervals comparing absolute prevalence error with the unweighted reference crossed zero. The prevalence analysis therefore did not support a clear condition-level advantage.

These quantities describe the pixel-area composition of the predicted foreground and should not be interpreted as percentages of positive tumor nuclei, Allred scores, H-scores, or slide-level ER status. Detailed prevalence results are shown in Supplementary Figure S8.

#### 4.5.3. Predictive Entropy, Ensemble Disagreement, and Error Ranking

Error-ranking behavior also depended on the evaluated pixel scope. For all-pixel entropy, unweighted random training achieved an AUROC of 0.913930 (95% source-group bootstrap interval [0.905458,0.922391]). Each weighted or minority-sensitive condition had a lower paired all-pixel entropy–error AUROC; the paired bootstrap differences relative to the unweighted reference ranged from −0.002853 to −0.005376, with all corresponding intervals below zero.

Within true foreground, the ordering reversed. The unweighted entropy–error AUROC was 0.779286, whereas the five weighted or minority-sensitive conditions ranged from 0.807395 to 0.815664. Weighted adaptive AMC produced the highest numerical true-foreground entropy–error AUROC of 0.815664, corresponding to a paired improvement of 0.036378 (95% source-group bootstrap interval [0.027135,0.045782]) relative to unweighted training.

True-foreground four-model disagreement showed a similar pattern. The variation-ratio error-ranking AUROC increased from 0.670328 for unweighted training to 0.704428 for weighted adaptive AMC, a paired difference of 0.034100 ([0.016417,0.053584]). In contrast, all-pixel variation-ratio differences were small, and their paired bootstrap intervals crossed zero.

When entropy was recalculated from probabilities renormalized over C1–C4 within true foreground, AUROCs were high for all conditions (0.915259 to 0.919204), but every paired comparator-versus-unweighted bootstrap interval crossed zero. Thus, the clearest scope-dependent difference arose when the original five-class entropy or four-model disagreement was evaluated on reference-foreground pixels.

**Figure 5:**
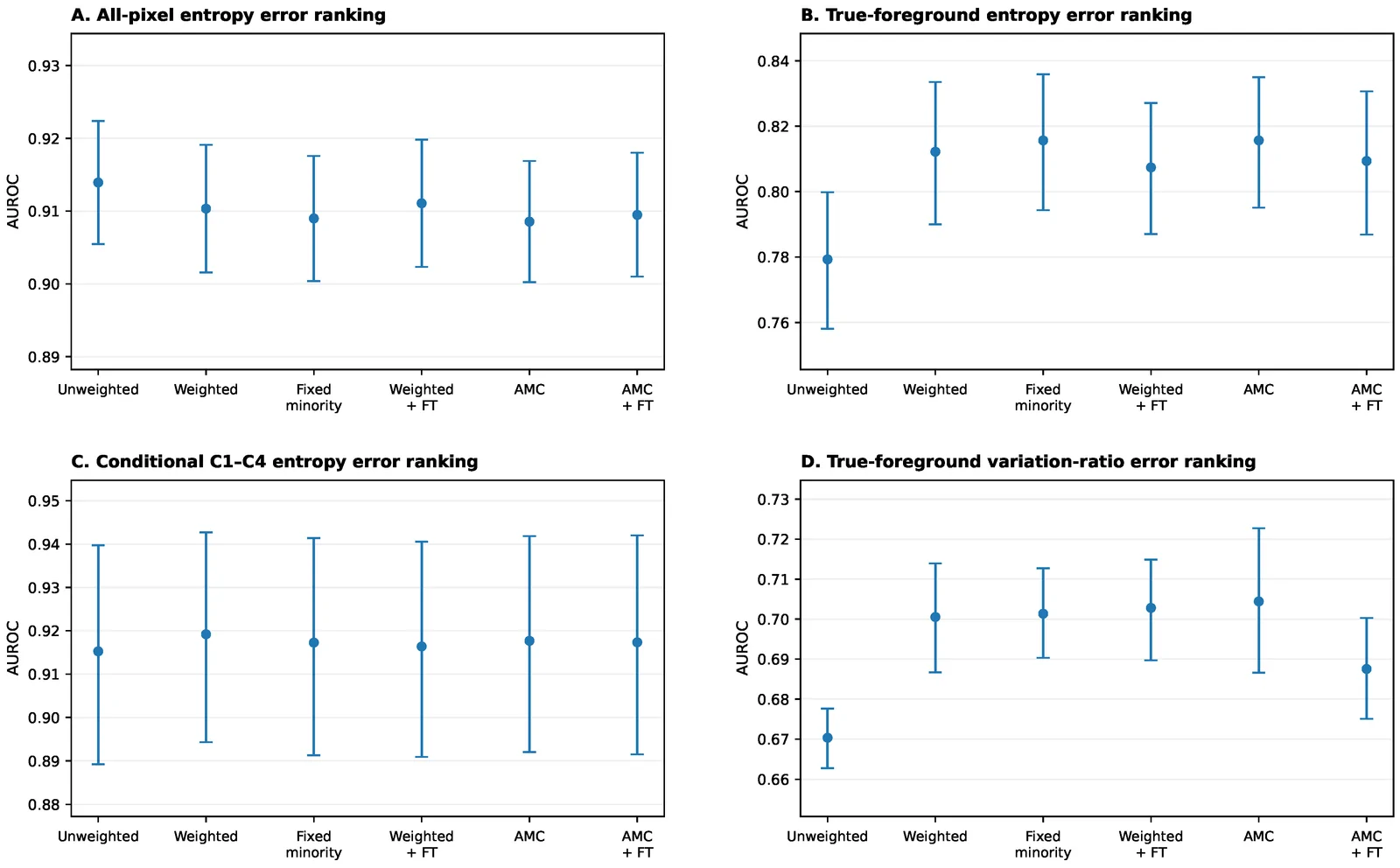
Error-ranking performance across the six source-group-preserving outer-test conditions using predictive entropy and four-model variation ratio. AUROC measures how well each score ranks incorrect pixels above correct pixels; higher values indicate better error ranking. These measures are distinct from probability calibration and should not be interpreted as validated clinical confidence or automated failure detection.

Figure 5 summarizes the uncertainty- and disagreement-based error-ranking results.

The source-group-resampling sensitivity analysis therefore suggested a consistent scope-dependent pattern: unweighted training was more favorable for global probability quality and all-pixel entropy-based error ranking, whereas weighted strategies improved selected probability-quality and error-ranking measures after conditioning on true foreground. These supportive bootstrap intervals were not multiplicity-controlled confirmatory tests and should not be interpreted as patient-level confidence intervals.

For the uncertainty/error-ranking endpoints, formal paired five-outer-fold comparisons were also performed using the same reference-versus-comparator framework as the primary analysis. No comparison reached p<0.05 before or after Holm adjustment; the minimum raw exact *p*-value was 0.0625, and the minimum Holm-adjusted value was 0.3125. Accordingly, the post hoc probability and error-ranking results are interpreted as scope-dependent supporting evidence rather than as evidence that one training strategy is uniformly superior.

Reliability diagrams, all-condition error-ranking summaries, class-specific calibration results, and detailed prevalence analyses are provided in Supplementary Figures S6–S8 and S18 and the corresponding Supplementary tables. None of the probability, entropy, or disagreement measures was interpreted as a validated clinical confidence estimate or as evidence of reliable automated error detection.

### 4.6. Supporting Development-Stage, Complexity, and Qualitative Analyses

Earlier component ablations, architecture comparisons, Focal–Tversky coefficient-sensitivity experiments, ordinal-loss variants, and C4-protective sampling analyses were performed during model development under the earlier non-nested five-fold training–validation protocol. These experiments are therefore retained as supporting development-stage evidence rather than as estimates of performance from the final source-group-preserving outer evaluation.

The development-stage experiments informed the selection of the ResUNet-DS backbone and the AMC and Focal–Tversky configurations subsequently included in the controlled six-condition comparison. Their validation results were not combined numerically with the final outer-test results and were not used to support claims of superiority in the source-group-preserving evaluation. Complete development-stage tables, figures, and configuration details are provided in the Supplementary Materials with their evaluation status explicitly identified.

At the individual-model level, the six primary conditions shared the same ResUNet-DS inference architecture, containing approximately 8.17 million trainable parameters. AMC modified training-crop selection and Focal–Tversky modified the training objective only; neither introduced an additional inference module. Supporting single-model computational profiling was performed during development, but those measurements should not be interpreted as the deployment cost of the final outer-test ensemble.

Each final outer-test prediction was generated from four independently selected inner models. Consequently, inference for one condition and outer fold required four model forward passes followed by arithmetic averaging of the four probability maps and pixel-wise argmax prediction. End-to-end latency, memory consumption, throughput, and energy use of this complete four-model ensemble were not measured. Detailed single-model profiling assumptions and architecture-level comparisons are therefore retained in the Supplementary Materials as supporting implementation characteristics.

**Figure 6:**
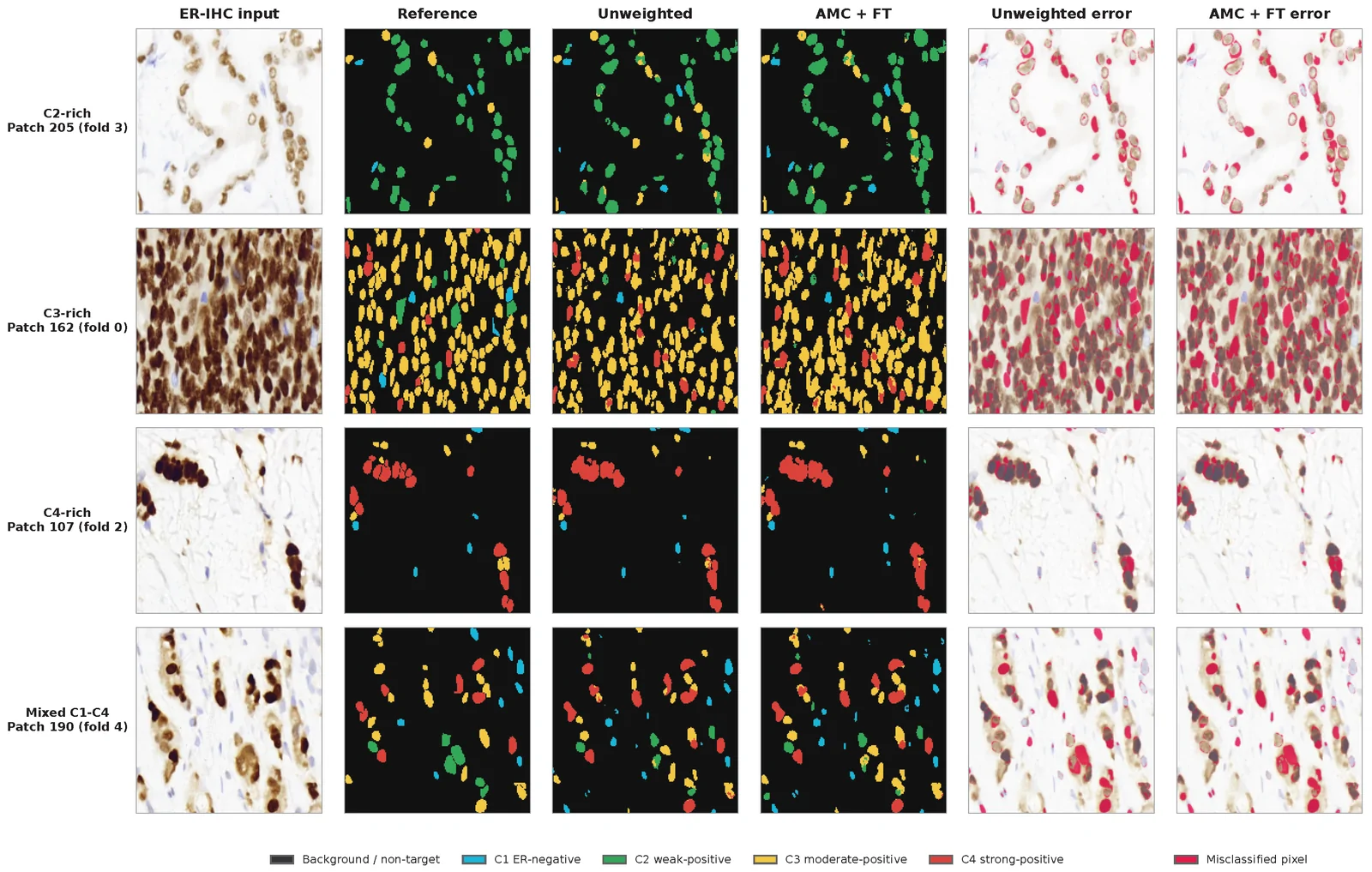
Representative qualitative examples from the frozen source-group-preserving outer-test predictions, including C2-rich, C3-rich, C4-rich, and mixed foreground cases from different outer folds. The examples illustrate spatial agreement and characteristic errors, including foreground omission, false foreground, fragmentation of positive-intensity regions, and confusion between neighboring C1–C4 categories. These examples are provided for qualitative interpretation only and were not used as independent statistical evidence.

Qualitative analysis was additionally performed using frozen source-group-preserving outer-test predictions. Representative cases were selected to illustrate C2-rich, C3-rich, C4-rich, and mixed foreground compositions across different outer folds rather than to identify best-performing examples. Figure 6 compares the input image, reference mask, ensemble prediction, and associated model-behavior summaries for these cases.

The qualitative examples demonstrate that high aggregate overlap can coexist with local errors in less-prevalent positive-intensity regions and at boundaries between neighboring expression categories. Probability, entropy, disagreement, and ordinal-error visualizations were interpreted as model-behavior summaries only; they were not treated as pathologist-replacement tools, automated quality-control systems, or evidence of clinical validation.

### 4.7. External ER-IHC Expression-Ordering Consistency

The IHC4BC experiment was retained as a supporting external-domain expression-ordering analysis. Compatible pixel-level C1–C4 reference masks were unavailable; therefore, model-derived spatial summaries were compared with independently available DAB-derived variables rather than evaluated as direct segmentation predictions.

The analysis had been performed during model development using a previously selected five-model AMC-Focal ResUNet-DS ensemble. This ensemble was distinct from the four-model inner ensembles used in the final source-group-preserving six-condition comparison. The IHC4BC experiment was not repeated across the six primary conditions and was not used to select or rank those conditions.

The development-stage ensemble was applied without retraining to 1226 ER-IHC images from seven patients. At the image level, predicted C3 area ratio showed a strong monotonic association with strong-DAB ratio (ρ=0.9674). The combined predicted C2–C4 area ratio was also strongly associated with mean DAB (ρ=0.9230) and ER-positive DAB ratio (ρ=0.9040).

Because the 1226 images originated from only seven patients, image observations were not interpreted as biologically independent replicates. Within-patient Spearman correlations between predicted C3 area ratio and strong-DAB ratio had a median of 0.9518 and ranged from 0.3796 to 0.9806, indicating that the strength of the association varied substantially among patients.

Predicted C4 occupied a substantially narrower dynamic range than C3. The mean predicted area ratios were 0.00862 for C3 and 0.00037 for C4, and predicted C3 and C4 were absent from 45.5% and 51.1% of images, respectively. This restricted C4 range limited the extent to which predicted C4 area could track between-image variation in strong-DAB measurements.

These results support expression-ordering consistency only. They do not establish equivalence between internal C3 and the external strong-DAB category, nor do they constitute external pixel-level segmentation validation, Allred agreement, H-score agreement, slide-level ER classification, or patient-level diagnostic performance. A summary of the supporting IHC4BC expression-ordering analysis is provided in Table 8.

The exploratory HER2-SISH40x analysis is reported in the Supplementary Materials as a cross-marker and cross-stain stress test. Because the resource represents a different biomarker, staining process, and label system, its categories were not mapped to the internal ER-IHC C1–C4 classes. No HER2 classification, amplification-scoring, or segmentation-performance claim was made. The analysis therefore provides descriptive evidence of model behavior under substantial domain shift only and is not interpreted as external ER-IHC validation or as evidence that the C1–C4 semantics transfer across biomarkers.

## 5. Discussion

This study evaluated whether class weighting, minority-focused crop sampling, adaptive minority curriculum (AMC), and Focal–Tversky optimization improved ordered ER-IHC segmentation under a source-group-preserving nested design. The principal finding was that the simplest unweighted random-training condition remained numerically strongest for all five primary aggregate endpoints, achieving mean foreground Dice of 0.7479, mean C2–C4 Dice of 0.7098, mean foreground IoU of 0.6074, foreground quadratically weighted kappa of 0.9761, and foreground-ordinal MAE of 0.0446. Among the five weighted or minority-sensitive conditions, weighted random training was numerically strongest on the same endpoints, but it did not exceed the unweighted reference.

The central result is therefore not that increasingly elaborate imbalance handling improved segmentation, but that the additional training complexity did not improve aggregate source-group-preserving outer-fold performance. This is notable because the foreground was strongly imbalanced: C1 ER-negative dominated, whereas C2–C4 were substantially less prevalent. Severe imbalance alone therefore did not imply that stronger weighting or minority-focused sampling would improve overall performance in this setting.

Class-specific behavior was nevertheless heterogeneous. Weighted random training achieved a slightly higher numerical C2 Dice than unweighted training (0.5994 versus 0.5957), despite lower aggregate C2–C4 Dice and lower overall foreground overlap. Thus, a local improvement in one less-prevalent class did not translate into better segmentation overall. This is consistent with the different mechanisms of the interventions: class weighting modifies relative loss contribution, minority-focused cropping modifies the spatial distribution of training examples, AMC adapts that crop composition using inner-validation minority performance, and Focal–Tversky alters the relative contribution of selected foreground errors. Their effects need not be additive and may redistribute performance among classes.

The development-stage experiments remain useful for understanding these mechanisms but should be interpreted separately from the final outer evaluation. Earlier architecture comparisons, loss ablations, Focal–Tversky sensitivity analyses, ordinal-loss experiments, and sampling variants informed the configurations tested here, but they were conducted during model development on the same resource. Their results therefore provide supporting evidence rather than independent confirmation of superiority.

Formal statistical inference was limited by the five paired outer folds. The final analysis comprised five predefined endpoints and five comparator-versus-unweighted contrasts, yielding 25 paired tests. Exact Wilcoxon *p*-values ranged from 0.0625 to 0.125, and none reached p<0.05; after Holm adjustment within each endpoint, all adjusted values were 0.3125. With only five non-zero paired differences, the smallest attainable two-sided exact *p*-value was 0.0625. Accordingly, the data do not provide multiplicity-adjusted evidence of superiority for any weighted or minority-sensitive condition.

The direction of the primary differences was nonetheless consistent. Unweighted training exceeded every comparator in all five folds for mean foreground Dice, mean C2–C4 Dice, and mean foreground IoU. The complementary 20,000-replicate paired source-group bootstrap preserved the same overall direction across all five primary endpoints and all five comparators. These intervals suggest that the observed ordering was not driven by only a few recovered-source groups, but they remain supportive sensitivity analyses rather than multiplicity-controlled confirmatory tests or patient-level confidence intervals.

The probability analyses revealed a different, scope-dependent pattern. Unweighted training provided the most favorable global probability quality over all pixels and for foreground-versus-background prediction, consistent with its stronger hard-segmentation performance. In contrast, after restricting evaluation to reference-foreground pixels, all weighted or minority-sensitive conditions improved the true-foreground multiclass Brier score relative to unweighted training, with weighted adaptive AMC giving the lowest numerical value. Weighted random training also performed best for the conditional C2–C4-versus-C1 probability task and showed the clearest class-specific Brier improvements for C1 and C2.

These findings demonstrate why global and conditional probability analyses should not be conflated. True-foreground evaluation asks how probability is distributed among expression categories after foreground location is already known; it does not assess whether the model detected foreground correctly in the first place. Conversely, all-pixel metrics are strongly influenced by the foreground-versus-background task. Imbalance correction can therefore improve conditional probability behavior without improving global segmentation, consistent with previous observations that class rebalancing can affect discrimination and calibration differently [26].

The prevalence analysis similarly showed no uniform advantage. Weighted random training produced the smallest numerical absolute error in the predicted C2–C4 fraction within foreground, but source-group bootstrap intervals for signed bias included zero for every condition, and paired intervals for absolute prevalence error crossed zero. These values describe pixel-area composition only and should not be interpreted as percentages of ER-positive tumor nuclei or established clinical scoring variables.

Error-ranking behavior was also scope-dependent. Unweighted training showed the strongest all-pixel entropy-based error ranking, whereas weighted conditions performed better when evaluation was restricted to true foreground. Weighted adaptive AMC produced the highest numerical true-foreground entropy- and variation-ratio-based error-ranking AUROCs. However, conditional C1–C4 entropy differences were small, their paired bootstrap intervals crossed zero, and the exploratory fold-level comparisons did not remain significant after Holm adjustment. Predictive entropy and variation ratio therefore remain descriptive model-behavior measures rather than validated clinical uncertainty estimates. Error-ranking AUROC indicates only how well incorrect pixels are ordered above correct pixels and does not establish calibrated failure probabilities or reliable automated error detection [27].

The ordinal metrics also have a specific boundary. Quadratically weighted kappa and ordinal MAE were calculated from the C1–C4 foreground submatrix, so foreground-to-background and background-to-foreground errors were outside these summaries and were captured instead by the segmentation and foreground-detection analyses. High foreground-ordinal agreement therefore reflects the ordering of retained foreground classifications rather than error-free foreground detection.

Clinical interpretation should remain similarly restricted. C1–C4 represent ER-negative, weak-positive, moderate-positive, and strong-positive foreground categories, while background/non-target is a separate label. Their pixel maps preserve spatial information about the supplied staining categories but are not validated Allred scores, H-scores, percentages of positive tumor nuclei, slide-level ER classifications, or treatment-decision variables. Translation to such endpoints would require tumor-cell identification, predefined whole-slide aggregation procedures, and appropriate pathologist-based clinical reference standards.

Annotation provenance is stronger than could be inferred from the patch archive alone. The expanded dataset publication describes 44 ER-IHC whole-slide images with five ROIs per slide and reports patch-wise review of segmentation and four-category assignments by two collaborating pathologists, with 110 ROIs assigned to each reviewer. The present study nevertheless used the distributed masks as fixed reference annotations and did not perform independent re-annotation, consensus adjudication, or a new interobserver-agreement study. Performance therefore represents agreement with the supplied references rather than with a prospectively generated consensus standard.

AMC and Focal–Tversky also did not alter the individual ResUNet-DS inference architecture, which contained approximately 8.17 million trainable parameters. Final predictions, however, were generated by four selected inner models per condition and outer fold. Thus, the final ensemble required four forward passes and probability averaging, and earlier single-model profiling should not be interpreted as the deployment cost of the complete ensemble.

The supporting IHC4BC analysis provides expression-ordering evidence rather than direct external segmentation validation. A previously selected development-stage five-model AMC-Focal ensemble showed strong associations between predicted C3 area and strong-DAB ratio (ρ=0.9674) and between predicted C2–C4 area and both mean DAB (ρ=0.9230) and ER-positive DAB ratio (ρ=0.9040). However, IHC4BC contained no compatible C1–C4 pixel masks, included only seven patients, and used a different development-stage ensemble from the final grouped comparison. Accordingly, these associations neither validate the final condition ranking nor establish external segmentation accuracy, Allred or H-score agreement, slide-level ER classification, or clinical generalizability. The HER2-SISH40x experiment is still more distant from the primary task and is interpreted only as an exploratory cross-marker and cross-stain stress test.

Overall, this single-resource study does not support the hypothesis that increasingly complex imbalance-handling strategies improve aggregate ordered ER-IHC segmentation over unweighted random training. The weighted strategies nevertheless altered probability quality and error-ranking behavior within true foreground. The findings therefore emphasize the importance of controlled grouped evaluation and of separating hard segmentation, probability quality, prevalence, and uncertainty-related endpoints when assessing imbalance-sensitive methods in computational pathology.

### Limitations and Future Directions

Several limitations constrain the interpretation of these findings. First, the study used only 220 512×512 patches from a single public ER-IHC resource. Provenance auditing recovered 44 five-patch source groups consistent with the published 44-slide, five-ROI-per-slide construction, and these groups were kept intact across partitions. However, the distributed patch package did not provide an authoritative mapping that independently verified each recovered identifier as a patient, specimen, tissue block, or whole-slide identifier. The evaluation therefore establishes source-group-preserving rather than independently verified patient-level independence.

Second, the grouped nested design constitutes strengthened internal evaluation rather than a completely independent confirmatory study. Training, AMC updates, and checkpoint selection were confined to the inner development partitions, but the architecture, six conditions, and several fixed hyperparameters were informed by earlier development using the same 220-patch resource. The final results should therefore be considered a rigorous internal re-evaluation rather than evidence from an independently developed and tested system.

Third, formal inference was based on only five paired outer folds. Exact Wilcoxon testing consequently had coarse resolution, and no comparison was significant after Holm adjustment. The source-group bootstrap provided useful sensitivity intervals but did not create additional independent biological units. Larger datasets with biologically independent test units are required for more precise and adequately powered comparisons.

Fourth, although the source publication reports patch-wise review by two collaborating pathologists, the present study did not independently re-annotate the images, perform new consensus adjudication, or measure interobserver agreement for the exact distributed masks. Model performance should therefore be interpreted against the supplied reference annotations rather than against a prospective multi-pathologist consensus.

Fifth, direct external validation remained unavailable. IHC4BC provided DAB-derived variables rather than compatible C1–C4 segmentation masks and included only seven patients, while HER2-SISH40x represented a different biomarker, staining process, and label system. Neither resource therefore established independent external accuracy for the five-class ER-IHC segmentation task.

Sixth, the study was restricted to image patches. Whole-slide inference, tumor-region and tumor-nucleus identification, positive-tumor-cell proportion, Allred and H-score calculation, slide-level ER status, clinical reporting, and treatment decisions were outside the study scope. Probability-quality, calibration, prevalence, entropy, disagreement, and error-ranking analyses were also post hoc and lacked an independent calibration cohort. Furthermore, the latency, memory, throughput, and energy requirements of the complete four-model ensemble were not measured.

Future work should therefore use datasets with authoritative patient, specimen, whole-slide, and source-region identifiers and should separate model development from an untouched, preferably multi-center, external cohort with harmonized ER-IHC annotations. Training conditions, checkpoint criteria, endpoints, and statistical comparisons should be prespecified before external testing.

Annotation studies should document tumor-region and tumor-nucleus criteria, staining-intensity definitions, pathologist review, adjudication, and interobserver agreement. Whole-slide studies should then define how spatial C1–C4 predictions are aggregated and evaluate their relationship with clinically established quantities such as positive tumor-cell proportion, staining intensity, Allred score, H-score, and slide-level ER status.

Probability calibration and uncertainty-based error detection should also be evaluated prospectively on independent data rather than inferred only from retrospective pixel-level analyses. Finally, the complete four-model ensemble should be profiled directly, and independently evaluated approaches such as single-model retraining, model distillation, or weight averaging could be explored if comparable predictive behavior can be preserved with lower deployment cost.

## 6. Conclusions

This study compared six class-weighting, crop-sampling, and loss configurations for ordered ER-IHC segmentation under a source-group-preserving nested evaluation. Unweighted random training achieved the most favorable numerical mean for all five primary endpoints, including foreground Dice of 0.7479, C2–C4 Dice of 0.7098, foreground IoU of 0.6074, foreground quadratically weighted kappa of 0.9761, and foreground-ordinal MAE of 0.0446. Weighted random training was the strongest weighted/minority-sensitive condition but did not exceed the unweighted reference. None of the 25 paired outer-fold comparisons reached p<0.05, and none was significant after Holm adjustment; the paired source-group bootstrap preserved the same overall direction.

Post hoc analyses showed scope-dependent behavior. Unweighted training provided stronger global probability quality and all-pixel entropy-based error ranking, whereas weighted strategies improved selected probability-quality and error-ranking measures within true foreground. These differences did not translate into superior aggregate segmentation and should not be interpreted as validated clinical confidence or automated failure detection.

The supporting IHC4BC analysis demonstrated expression-ordering consistency with DAB-derived measurements but did not provide direct external C1–C4 segmentation validation, while HER2-SISH40x remained an exploratory cross-marker and cross-stain stress test.

Overall, increasingly complex imbalance-handling strategies did not improve aggregate ER-IHC segmentation over the simpler unweighted reference in this single-resource evaluation. The findings should therefore be interpreted as preliminary methodological evidence rather than patient-level, whole-slide, externally validated, or clinically deployable performance. Future work requires biologically identified test units, harmonized multi-center external annotations, whole-slide evaluation, and independent pathologist-based validation.

## Figures and Tables

**Table 1:** Pixel-level class distribution of the 220 cleaned reference masks.

Class ID	Annotation Meaning	Total Pixels	Pixel Ratio	Patches Present
0	Background/non-target	44,073,570	0.7642	220
1	C1 ER-negative	10,208,024	0.1770	218
2	C2 weak-positive	886,332	0.0154	127
3	C3 moderate-positive	1,496,739	0.0260	93
4	C4 strong-positive	1,007,015	0.0175	65

**Table 2:** Composition of the five source-group-preserving outer-test folds. Class-presence counts indicate the number of patches containing at least one reference pixel from the corresponding class.

Outer Fold	Source Groups	Test Patches	C2-Present Patches	C3-Present Patches	C4-Present Patches
0	9	45	29	21	15
1	9	45	24	15	12
2	9	45	27	22	14
3	9	45	24	17	13
4	8	40	23	18	11

**Table 3:** Common training configuration used for the six primary conditions.

Setting	Value
Training duration	120 epochs per inner model
Inner-training crop size	320×320 pixels
Inner-validation input	Complete 512×512 patches without augmentation
Training batch size	2
Validation batch size	1
Optimizer	AdamW
Initial learning rate	$3\times 10^{-4}$
Weight decay	$1\times 10^{-4}$
Gradient clipping	Maximum norm of 1.0
Data-loading workers	2
Mixed precision	Enabled when CUDA was available
Base random seed	42
Split-specific seed	$s_{o,v}=42+100o+v$

**Table 4:** Controlled training components defining the six primary experimental conditions.

Condition	Class Weighting	Crop-Selection Strategy	Focal–Tversky
Unweighted random	No	Random	No
Weighted random	Yes	Random	No
Weighted fixed-minority	Yes	Fixed-minority probability 0.25	No
Weighted random + Focal–Tversky	Yes	Random	Yes
Weighted adaptive AMC	Yes	Adaptive minority curriculum	No
Weighted adaptive AMC + Focal–Tversky	Yes	Adaptive minority curriculum	Yes

**Table 5:** Primary source-group-preserving nested outer-test results. Values are mean ± sample standard deviation across the five outer folds. Bold values indicate the numerically best mean in each column according to the direction of the metric and do not imply statistical significance. Higher values are better for Dice, IoU, and weighted kappa; lower ordinal MAE is better.

Condition	Mean Foreground Dice C1–C4	Mean Dice C2–C4	Mean Foreground IoU C1–C4	Foreground Quadratic Weighted Kappa	Foreground Ordinal MAE
Unweighted random	0.7479±0.0123	0.7098±0.0128	0.6074±0.0147	0.9761±0.0040	0.0446±0.0062
Weighted random	0.7397±0.0152	0.7013±0.0154	0.5961±0.0181	0.9748±0.0042	0.0463±0.0060
Weighted fixed-minority	0.7320±0.0125	0.6907±0.0133	0.5871±0.0167	0.9730±0.0055	0.0499±0.0092
Weighted random + Focal–Tversky	0.7342±0.0142	0.6934±0.0147	0.5900±0.0160	0.9734±0.0043	0.0488±0.0062
Weighted adaptive AMC	0.7355±0.0165	0.6954±0.0198	0.5915±0.0189	0.9743±0.0053	0.0472±0.0079
Weighted adaptive AMC + Focal–Tversky	0.7349±0.0090	0.6948±0.0091	0.5906±0.0116	0.9732±0.0051	0.0493±0.0080

**Table 6:** Paired outer-fold comparisons of each weighted or minority-sensitive condition against unweighted random training. Differences are comparator minus reference. Values in brackets are 95% *t*-intervals across the five paired outer-fold differences. $r_{\mathrm{rb}}$ denotes the rank-biserial effect size. Holm adjustment was performed separately within each endpoint across the five comparator-versus-reference tests.

Endpoint	Comparator	Mean Difference [95% CI]	$d_{z}$	$r_{\mathrm{rb}}$	Holm *p*
Mean foreground Dice	Weighted random	−0.0081[−0.0126,−0.0036]	−2.24	−1.00	0.3125
	Weighted fixed-minority	−0.0158[−0.0239,−0.0078]	−2.44	−1.00	0.3125
	Weighted random + FT	−0.0136[−0.0218,−0.0055]	−2.09	−1.00	0.3125
	Weighted adaptive AMC	−0.0123[−0.0189,−0.0058]	−2.34	−1.00	0.3125
	Weighted adaptive AMC + FT	−0.0129[−0.0215,−0.0043]	−1.87	−1.00	0.3125
Mean C2–C4 Dice	Weighted random	−0.0085[−0.0126,−0.0044]	−2.57	−1.00	0.3125
	Weighted fixed-minority	−0.0191[−0.0301,−0.0081]	−2.16	−1.00	0.3125
	Weighted random + FT	−0.0164[−0.0260,−0.0068]	−2.12	−1.00	0.3125
	Weighted adaptive AMC	−0.0144[−0.0247,−0.0040]	−1.73	−1.00	0.3125
	Weighted adaptive AMC + FT	−0.0150[−0.0245,−0.0055]	−1.96	−1.00	0.3125
Mean foreground IoU	Weighted random	−0.0113[−0.0177,−0.0048]	−2.16	−1.00	0.3125
	Weighted fixed-minority	−0.0203[−0.0302,−0.0103]	−2.53	−1.00	0.3125
	Weighted random + FT	−0.0174[−0.0271,−0.0077]	−2.22	−1.00	0.3125
	Weighted adaptive AMC	−0.0159[−0.0233,−0.0084]	−2.65	−1.00	0.3125
	Weighted adaptive AMC + FT	−0.0168[−0.0283,−0.0053]	−1.81	−1.00	0.3125
Weighted kappa	Weighted random	−0.0013[−0.0021,−0.0004]	−1.89	−1.00	0.3125
	Weighted fixed-minority	−0.0031[−0.0055,−0.0006]	−1.56	−1.00	0.3125
	Weighted random + FT	−0.0027[−0.0052,−0.0001]	−1.29	−0.87	0.3125
	Weighted adaptive AMC	−0.0018[−0.0039,+0.0004]	−1.03	−0.87	0.3125
	Weighted adaptive AMC + FT	−0.0029[−0.0050,−0.0008]	−1.68	−1.00	0.3125
Ordinal MAE	Weighted random	+0.0017[+0.0006,+0.0028]	+1.87	+1.00	0.3125
	Weighted fixed-minority	+0.0053[+0.0009,+0.0096]	+1.49	+1.00	0.3125
	Weighted random + FT	+0.0042[−0.0005,+0.0088]	+1.12	+0.87	0.3125
	Weighted adaptive AMC	+0.0026[−0.0005,+0.0057]	+1.04	+0.87	0.3125
	Weighted adaptive AMC + FT	+0.0046[+0.0016,+0.0077]	+1.90	+1.00	0.3125

**Table 7:** Brier-score summaries from the frozen outer-test probabilities. The four columns evaluate different probability questions and should not be interpreted interchangeably. Lower values are better. Bold values indicate the lowest numerical Brier score within each column.

Condition	Five-Class All Pixels	Five-Class True Foreground	Foreground vs. Background All Pixels	C2–C4 vs. C1 Within True Foreground
Unweighted random	**0.104940**	0.240767	**0.045823**	0.012931
Weighted random	0.115433	0.188662	0.051523	**0.012368**
Weighted fixed-minority	0.116686	0.190030	0.051816	0.013238
Weighted random + Focal–Tversky	0.114836	0.202891	0.050740	0.013108
Weighted adaptive AMC	0.116685	**0.187194**	0.052043	0.012867
Weighted adaptive AMC + Focal–Tversky	0.116013	0.196475	0.051271	0.013501

**Table 8:** Summary of the supporting IHC4BC ER-IHC expression-ordering analysis.

Dataset	Analysis Level	Principal Evidence	Interpretive Boundary
IHC4BC ER-IHC (n=1226; seven patients)	Image-level and within-patient association	Predicted C3 ratio versus strong-DAB ratio: ρ=0.9674; predicted C2–C4 ratio versus mean DAB: ρ=0.9230; predicted C2–C4 ratio versus ER-positive DAB ratio: ρ=0.9040.	Expression-ordering consistency only. Compatible C1–C4 reference masks, a validated class-to-DAB mapping, direct clinical-score references, and a sufficiently large independent patient cohort were unavailable.

## Data Availability

The internal ER-IHC dataset used in this study is available from IEEE DataPort: UMMC ER-IHC Breast Histopathology Whole-Slide Image and Allred Score [36]. Access to this dataset is subject to the terms and conditions of IEEE DataPort. The external IHC4BC dataset used for the supporting ER-IHC expression-ordering analysis is publicly available from the IHC4BC resource described by Akbarnejad et al. [6]. The HER2-SISH40x dataset used for cross-marker and cross-stain robustness analysis is described by Rehman et al. [7]. The compact external subsets, derived prediction summaries, and report figures generated during this study are available from the corresponding author upon reasonable request, subject to the access conditions and reuse terms of the original datasets. The source code and configuration files used for preprocessing, model training, component ablation, controlled imbalance experiments, comparative evaluation, uncertainty and calibration analysis, external expression-ordering analysis, supplementary cross-marker stress testing, and figure generation are maintained in a GitHub repository. The repository will be made publicly available upon acceptance of the manuscript at: https://github.com/Arefin-Saiful/ER_IHC_Segmentation accessed on 24 August 2026. Access to the datasets remains subject to the terms and availability conditions of the original data providers.

## Supplementary Materials

The following supporting information can be downloaded at: https://www.mdpi.com/article/10.3390/diagnostics16172752/s1, Table S1: Primary source-group-preserving nested outer-test results; Table S2: Paired source-group-preserving outer-fold comparisons; Table S3: Source-group-resampling sensitivity analysis; Table S4: Pooled Brier scores across the four predefined probability scopes; Table S5: Pooled negative log-likelihood and expected calibration error across the four probability scopes; Table S6: Pooled class-specific calibration within true foreground; Table S7: C2–C4 fraction within foreground; Table S8: Predictive entropy, ensemble disagreement, and error-ranking analysis; Table S9: Exploratory HER2-SISH40x cross-marker and cross-stain stress test; Figure S1: Pixel-level class distribution across the five source-group-preserving outer-test folds; Figure S2: Foreground and positive-intensity balance across the five source-group-preserving outer-test folds; Figure S3: Patch-level class presence across the five source-group-preserving outer-test folds; Figures S4–S21: Supporting architecture comparisons, calibration analyses, uncertainty analyses, development-stage experiments, external-domain expression-ordering analyses, and exploratory cross-marker and cross-stain stress-test results. The Supplementary Materials are provided as a separate file and include detailed six-condition outer-test results; paired fold-level statistical comparisons and source-group-resampling analyses; class-specific and scope-dependent probability-quality, prevalence, entropy, reliability, and ensemble-disagreement analyses; outer-fold composition summaries; supporting non-nested development-stage architecture, ablation, sensitivity, ordinal, and training analyses; IHC4BC expression-ordering analyses; and an exploratory HER2-SISH40x cross-marker and cross-stain stress test.
