# Supplementary material for: Toward AI-Assisted Precision Diagnostics in Breast Cancer: Source-Group-Preserving Evaluation of Class-Imbalance Strategies for Ordered ER-IHC Segmentation
Source: Diagnostics (Basel). 2026 Aug 27;16(17):2752. doi: 10.3390/diagnostics16172752 (PMC13565420; doi:10.3390/diagnostics16172752)
Supplement: Supplementary file 1 [file diagnostics-16-02752-s001.zip › diagnostics-4428665-supplementary.pdf]

Supplementary Material

Toward AI-Assisted Precision Diagnostics in Breast Cancer: Source-Group-Preserving Evaluation of Class-Imbalance Strategies for Ordered ER-IHC Segmentation

Md Saiful Arefin, Mohammad Saiful Islam, Md Serajun Nabi, Rashadul Islam Sumon, Maria Lapina, Vitalii Lapin, and Mohammed Muthanna

Scope and Evidence Hierarchy

The primary evidence in this supplement is derived from the source-group-preserving nested five-fold evaluation described in the main manuscript. Provenance auditing recovered 44 five-patch source groups from the 220-patch ER-IHC resource. These groups were assigned intact to outer folds containing 9, 9, 9, 9, and 8 source groups, corresponding to 45, 45, 45, 45, and 40 patches, respectively. Within each outer iteration, the remaining four grouped folds rotated once each as inner validation. Four independently selected inner models from the same condition were ensembled by arithmetic averaging of their per-pixel softmax probabilities on the untouched outer-test fold.

Six conditions were evaluated under the same ResUNet-DS inference architecture: unweighted random training, weighted random training, weighted fixed-minority sampling, weighted random training with Focal-Tversky, weighted adaptive minority curriculum (AMC), and weighted adaptive AMC with Focal-Tversky. The five outer folds were treated as paired cross-validation evaluation blocks for the formal condition-level comparisons; they are not independently verified biological units. Pixel- and patch-level pooled values were not treated as independent inferential observations. Paired source-group resampling was used separately as a supportive dependence-aware sensitivity analysis.

Architecture comparisons, component ablations, coefficient-sensitivity experiments, C4-protective sampling, ordinal variants, and associated learning curves were completed during the earlier non-nested model-development stage. They are retained below only as supporting development evidence and must not be combined with the final grouped outer-test estimates to support a superiority claim.

The IHC4BC analysis was performed during model development using a previously selected five-model AMC-Focal ResUNet-DS ensemble and is retained only as an external-domain expression-ordering analysis. The HER2-SISH40x analysis is an exploratory cross-marker and cross-stain stress test. Neither external resource provides compatible pixel-level C1–C4 reference masks; neither analysis constitutes direct external five-class segmentation validation, clinical-score validation, or confirmation of the final six-condition ranking.

Primary Source-Group-Preserving Outer-Test Results

Table S1: Primary source-group-preserving nested outer-test results. Values are mean ± sample standard deviation across the five outer folds. Bold values indicate the best numerical mean in each column and do not imply statistical significance. Higher values are better for Dice, IoU, and weighted kappa; lower ordinal MAE is better.

Condition	Mean foreground Dice C1–C4	Mean Dice C2–C4	Mean foreground IoU C1–C4	Foreground quadratic weighted kappa	Foreground ordinal MAE
Unweighted random	**0.7479 ± 0.0123**	**0.7098 ± 0.0128**	**0.6074 ± 0.0147**	**0.9761 ± 0.0040**	**0.0446 ± 0.0062**
Weighted random	0.7397 ± 0.0152	0.7013 ± 0.0154	0.5961 ± 0.0181	0.9748 ± 0.0042	0.0463 ± 0.0060
Weighted fixed-minority	0.7320 ± 0.0125	0.6907 ± 0.0133	0.5871 ± 0.0167	0.9730 ± 0.0055	0.0499 ± 0.0092
Weighted random + Focal-Tversky	0.7342 ± 0.0142	0.6934 ± 0.0147	0.5900 ± 0.0160	0.9734 ± 0.0043	0.0488 ± 0.0062
Weighted adaptive AMC	0.7355 ± 0.0165	0.6954 ± 0.0198	0.5915 ± 0.0189	0.9743 ± 0.0053	0.0472 ± 0.0079
Weighted adaptive AMC + Focal-Tversky	0.7349 ± 0.0090	0.6948 ± 0.0091	0.5906 ± 0.0116	0.9732 ± 0.0051	0.0493 ± 0.0080

Unweighted random training achieved the most favourable numerical mean for all five primary endpoints. Weighted random training was the strongest of the five weighted or minority-sensitive conditions on these same aggregate endpoints but did not exceed the unweighted reference. Class-specific behaviour was not identical to the aggregate ordering; for example, weighted random achieved a slightly higher numerical C2 Dice than unweighted training (0.5994 versus 0.5957), despite lower mean C2–C4 Dice and lower mean foreground overlap.

Paired Outer-Fold Comparisons

Table S2 reports all 25 predefined comparator-versus-unweighted-reference comparisons. The difference is comparator minus reference. Comparator fold wins and losses respect the direction of each endpoint (higher is better except for ordinal MAE). All rows had zero ties.

Table S2: Paired source-group-preserving outer-fold comparisons of each weighted or minority-sensitive condition against unweighted random training. Differences are comparator minus reference and brackets contain descriptive 95% t-intervals across the five paired fold differences. d_z is Cohen's paired standardised effect size and r_{rb} is the rank-biserial effect size. Holm adjustment was applied separately within each endpoint across the five comparator-versus-reference tests.

Endpoint	Comparator	Difference [95% CI]	d_z	r_{rb}	W/L	Raw p	Holm p
Mean foreground Dice	Weighted random	−0.0081 [−0.0126, −0.0036]-2.24		-1.00	0/5	0.0625	0.3125
	Weighted fixed-minority	−0.0158 [−0.0239, −0.0078]-2.44		-1.00	0/5	0.0625	0.3125
	Weighted random + FT	−0.0136 [−0.0218, −0.0055]-2.09		-1.00	0/5	0.0625	0.3125
	Weighted adaptive AMC	−0.0123 [−0.0189, −0.0058]-2.34		-1.00	0/5	0.0625	0.3125
	Weighted adaptive AMC + FT	−0.0129 [−0.0215, −0.0043]-1.87		-1.00	0/5	0.0625	0.3125
Mean C2–C4 Dice	Weighted random	−0.0085 [−0.0126, −0.0044]-2.57		-1.00	0/5	0.0625	0.3125
	Weighted fixed-minority	−0.0191 [−0.0301, −0.0081]-2.16		-1.00	0/5	0.0625	0.3125
	Weighted random + FT	−0.0164 [−0.0260, −0.0068]-2.12		-1.00	0/5	0.0625	0.3125
	Weighted adaptive AMC	−0.0144 [−0.0247, −0.0040]-1.73		-1.00	0/5	0.0625	0.3125
	Weighted adaptive AMC + FT	−0.0150 [−0.0245, −0.0055]-1.96		-1.00	0/5	0.0625	0.3125
Mean foreground IoU	Weighted random	−0.0113 [−0.0177, −0.0048]-2.16		-1.00	0/5	0.0625	0.3125
	Weighted fixed-minority	−0.0203 [−0.0302, −0.0103]-2.53		-1.00	0/5	0.0625	0.3125

Endpoint	Comparator	Difference [95% CI]	d_z	r_{rb}	W/L	Raw p	Holm p
	Weighted random + FT	-0.0174 [$-0.0271, -0.0077$]	-2.22	-1.00	0/5	0.0625	0.3125
	Weighted adaptive AMC	-0.0159 [$-0.0233, -0.0084$]	-2.65	-1.00	0/5	0.0625	0.3125
	Weighted adaptive AMC + FT	-0.0168 [$-0.0283, -0.0053$]	-1.81	-1.00	0/5	0.0625	0.3125
Weighted kappa	Weighted random	-0.0013 [$-0.0021, -0.0004$]	-1.89	-1.00	0/5	0.0625	0.3125
	Weighted fixed-minority	-0.0031 [$-0.0055, -0.0006$]	-1.56	-1.00	0/5	0.0625	0.3125
	Weighted random + FT	-0.0027 [$-0.0052, -0.0001$]	-1.29	-0.87	1/4	0.1250	0.3125
	Weighted adaptive AMC	-0.0018 [$-0.0039, +0.0004$]	-1.03	-0.87	1/4	0.1250	0.3125
	Weighted adaptive AMC + FT	-0.0029 [$-0.0050, -0.0008$]	-1.68	-1.00	0/5	0.0625	0.3125
Ordinal MAE	Weighted random	$+0.0017$ [$+0.0006, +0.0028$]	+1.87	+1.00	0/5	0.0625	0.3125
	Weighted fixed-minority	$+0.0053$ [$+0.0009, +0.0096$]	+1.49	+1.00	0/5	0.0625	0.3125
	Weighted random + FT	$+0.0042$ [$-0.0005, +0.0088$]	+1.12	+0.87	1/4	0.1250	0.3125
	Weighted adaptive AMC	$+0.0026$ [$-0.0005, +0.0057$]	+1.04	+0.87	1/4	0.1250	0.3125
	Weighted adaptive AMC + FT	$+0.0046$ [$+0.0016, +0.0077$]	+1.90	+1.00	0/5	0.0625	0.3125

None of the 25 exact two-sided Wilcoxon comparisons reached $p < 0.05$ before multiplicity correction. Raw exact p-values ranged from 0.0625 to 0.125, and every Holm-adjusted p-value was 0.3125. With five paired non-zero fold differences, the smallest attainable two-sided exact p-value was 0.0625. These fold-level tests therefore have limited inferential resolution and are not patient-level or whole-slide-level tests.

Source-Group-Resampling Sensitivity Analysis

A complementary paired bootstrap resampled recovered five-patch source groups with replacement within the five fixed outer folds. The same draw was applied across all six conditions. A total of 20,000 replicates were generated using seed 20260812.

Table S3: Pooled point estimates for unweighted random training and 95% source-group bootstrap sensitivity intervals. These intervals quantify sensitivity to recovered source-group composition and are not patient-level confidence intervals.

Endpoint	Pooled estimate	95% bootstrap interval
Mean foreground Dice	0.749400	[0.733602, 0.760310]
Mean C2–C4 Dice	0.711611	[0.690462, 0.725649]
Mean foreground IoU	0.608430	[0.591049, 0.621507]
Foreground weighted kappa	0.976364	[0.968295, 0.980784]
Foreground ordinal MAE	0.044264	[0.028918, 0.062116]

For all five primary endpoints and all five comparators, the paired bootstrap benefit intervals retained the direction favouring the unweighted reference and excluded zero. For example, weighted random minus unweighted was -0.009019 ($[-0.015026, -0.002708]$) for mean foreground Dice, -0.009777 ($[-0.017393, -0.001710]$) for mean C2–C4 Dice, and -0.012176 ($[-0.019063, -0.004605]$) for mean foreground IoU. These intervals were used only as supportive internal sensitivity summaries and not as additional multiplicity-controlled significance tests.

Post-Hoc Probability Quality, Prevalence, and Error Ranking

All analyses in this section were derived from the frozen source-group-preserving outer-test outputs. No temperature scaling, probability recalibration, threshold tuning, checkpoint replacement, model exclusion, or post-test model selection was performed. Pooled pixel-level metrics are descriptive summaries. Dependence-aware 95% sensitivity intervals were obtained from the same 20,000 paired source-group bootstrap draws described above.

Probability Quality Across Four Evaluation Scopes

Probability quality was evaluated for (i) five-class prediction over all pixels, (ii) the same five-class prediction restricted to reference-foreground pixels without renormalising away background probability, (iii) binary foreground-versus-background prediction over all pixels, and (iv) binary C2–C4-versus-C1 probability allocation within true foreground.

Table S4: Pooled Brier scores across the four predefined probability scopes. Lower values indicate better probability quality. Bold values mark the lowest numerical Brier score within each scope.

Condition	Five-class all pixels	Five-class true foreground	Foreground vs. background all pixels	C2–C4 vs. C1 within true foreground
Unweighted random	**0.104940**	0.240767	**0.045823**	0.012931
Weighted random	0.115433	0.188662	0.051523	**0.012368**
Weighted fixed-minority	0.116686	0.190030	0.051816	0.013238
Weighted random + FT	0.114836	0.202891	0.050740	0.013108
Weighted adaptive AMC	0.116685	**0.187194**	0.052043	0.012867
Weighted adaptive AMC + FT	0.116013	0.196475	0.051271	0.013501

Table S5: Pooled negative log-likelihood (NLL) and fixed 15-bin ECE across the four probability scopes. Lower values are better. ECE is reported as a descriptive, bin-dependent reliability summary and was not used as a stand-alone condition-ranking criterion.

	Five-class, all pixels		Five-class, true foreground		Foreground vs. background		C2–C4 vs. C1	
Condition	NLL	ECE_{15}	NLL	ECE_{15}	NLL	ECE_{15}	NLL	ECE_{15}
Unweighted random	0.182772	0.002535	0.433734	0.011610	0.151886	0.008048	0.045812	0.003999
Weighted random	0.197974	0.005754	0.342825	0.014370	0.168477	0.035794	0.043447	0.001625
Weighted fixed-minority	0.200982	0.005281	0.345930	0.013772	0.169688	0.037524	0.045936	0.002857
Weighted random + FT	0.197872	0.006353	0.366957	0.006197	0.166367	0.030323	0.046717	0.003706
Weighted adaptive AMC	0.200431	0.004154	0.340692	0.020619	0.170569	0.038168	0.044455	0.003206
Weighted adaptive AMC + FT	0.199552	0.005892	0.357258	0.008120	0.168331	0.033350	0.046264	0.004161

Unweighted random training gave the lowest all-pixel five-class Brier score and NLL and the lowest foreground-versus-background Brier score and NLL. In contrast, all weighted or minority-sensitive conditions reduced true-foreground five-class Brier score relative to unweighted training. Weighted adaptive AMC had the lowest numerical true-foreground Brier score, whereas weighted random had the lowest C2–C4-versus-C1 Brier score and NLL. This scope-dependent ordering does not imply that the weighted conditions improved global segmentation.

True-Foreground Class-Specific Calibration

For class-specific analysis within reference foreground, probabilities were renormalised over C1–C4 and one-versus-rest Brier score and fixed 15-bin ECE were calculated for each foreground category.

Table S6: Pooled class-specific calibration within true foreground. Each cell reports Brier score / fixed 15-bin ECE after renormalising probabilities over C1–C4. Lower values are better. These analyses are exploratory and were not part of the multiplicity-controlled primary endpoint family.

Condition	C1 ER-negative	C2 weak-positive	C3 moderate-positive	C4 strong-positive
Unweighted random	0.012931 / 0.003999	0.021349 / 0.007004	0.024979 / 0.008065	0.016351 / 0.004635
Weighted random	0.012368 / 0.001625	0.020062 / 0.004610	0.024678 / 0.003219	0.016758 / 0.002728
Weighted fixed-minority	0.013238 / 0.002857	0.021927 / 0.007754	0.026166 / 0.005996	0.017147 / 0.002201
Weighted random + FT	0.013108 / 0.003706	0.021406 / 0.009470	0.025922 / 0.004976	0.017501 / 0.005869
Weighted adaptive AMC	0.012867 / 0.003206	0.020764 / 0.007030	0.024731 / 0.003325	0.016624 / 0.002884
Weighted adaptive AMC + FT	0.013501 / 0.004161	0.021752 / 0.007242	0.025815 / 0.006441	0.017354 / 0.004263

Weighted random showed the clearest paired source-group Brier improvements for C1 and C2 relative to unweighted training: 0.000563 ($[0.000069, 0.001153]$) for C1 and 0.001287 ($[0.000521, 0.002184]$) for C2 when benefit was expressed as unweighted minus weighted random. The corresponding C3 and C4 intervals crossed zero. Paired class-specific ECE intervals also crossed zero and were interpreted descriptively.

Positive-Intensity Foreground Composition

The principal prevalence-preservation quantity was the hard-predicted fraction of C2–C4 pixels within predicted C1–C4 foreground, compared with the reference C2–C4 foreground fraction of 0.249306.

Table S7: Pooled C2–C4 fraction within foreground. Signed bias is predicted minus reference. Intervals are 95% source-group bootstrap sensitivity intervals for signed bias. Absolute error is the absolute signed bias. These quantities are pixel-area summaries, not percent-positive tumour-nucleus or clinical-score estimates.

Condition	Predicted C2–C4 fraction	Signed bias [95% CI]	Absolute error
Unweighted random	0.242948	$-0.006358\,[-0.014617, +0.001483]$	0.006358
Weighted random	0.251632	$+0.002326\,[-0.006439, +0.011350]$	**0.002326**
Weighted fixed-minority	0.256709	$+0.007403\,[-0.002057, +0.016531]$	0.007403
Weighted random + FT	0.257304	$+0.007999\,[-0.002560, +0.018171]$	0.007999
Weighted adaptive AMC	0.255965	$+0.006660\,[-0.004540, +0.016885]$	0.006660
Weighted adaptive AMC + FT	0.255405	$+0.006099\,[-0.002480, +0.015290]$	0.006099

Weighted random had the smallest numerical absolute error, but every condition's signed-bias interval included zero and every paired bootstrap interval comparing absolute prevalence error with the unweighted reference crossed zero. The prevalence analysis therefore did not establish a clear condition-level advantage.

Predictive Entropy, Ensemble Disagreement, and Error Ranking

Table S8: Pooled uncertainty- and disagreement-based error-ranking AUROC across the six source-group-preserving conditions. Higher AUROC indicates better ranking of incorrect pixels above correct pixels; it does not measure calibration or establish clinical confidence.

Condition	Entropy all pixels	Variation ratio all pixels	Entropy true foreground	Variation ratio true foreground	Conditional C1–C4 entropy
Unweighted random	0.913930	0.698095	0.779286	0.670328	0.915259
Weighted random	0.910336	0.696371	0.812191	0.700526	**0.919204**
Weighted fixed-minority	0.908994	**0.701010**	0.815644	0.701349	0.917273
Weighted random + FT	0.911077	0.697248	0.807395	0.702808	0.916378
Weighted adaptive AMC	0.908555	0.699421	**0.815664**	**0.704428**	0.917681
Weighted adaptive AMC + FT	0.909485	0.693496	0.809363	0.687534	0.917341

Unweighted random achieved an all-pixel entropy–error AUROC of 0.913930 with a 95% source-group bootstrap interval of $[0.905458, 0.922391]$. Each weighted or minority-sensitive condition had a lower paired all-pixel entropy–error AUROC. Within true foreground, the ordering reversed: weighted adaptive AMC achieved the highest numerical entropy–error AUROC (0.815664) and variation-ratio error-ranking AUROC (0.704428). Relative to unweighted training, the paired source-group bootstrap improvements were 0.036378 ($[0.027135, 0.045782]$) and 0.034100 ($[0.016417, 0.053584]$), respectively.

Conditional C1–C4 entropy–error AUROCs were high for all conditions (0.915259–0.919204), but every paired comparator-versus-unweighted bootstrap interval crossed zero. Formal paired outer-fold comparisons for the five error-ranking endpoints also yielded no raw or Holm-adjusted $p < 0.05$; the minimum raw exact p-value was 0.0625 and the minimum Holm-adjusted value was 0.3125. These analyses are exploratory and do not establish calibrated failure probabilities or reliable automated error detection.

Supplementary Figures

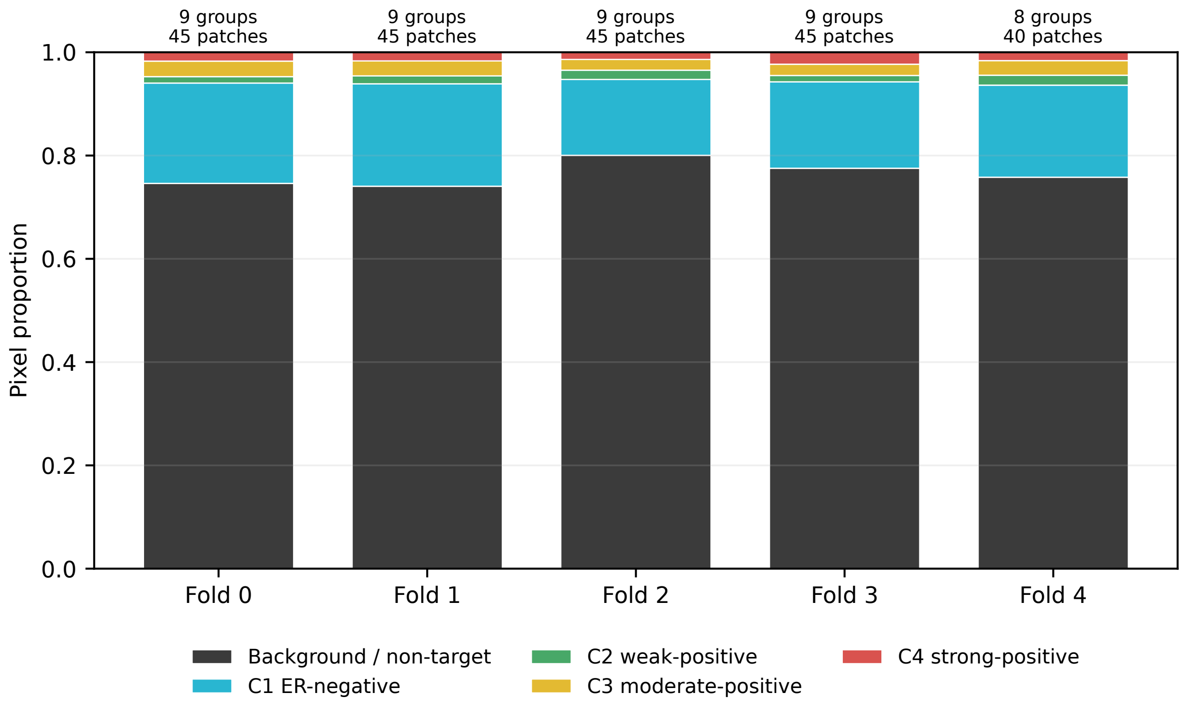

Figure S1: Pixel-level class distribution across the five source-group-preserving outer-test folds. The recovered five-patch source groups were kept intact during partitioning.

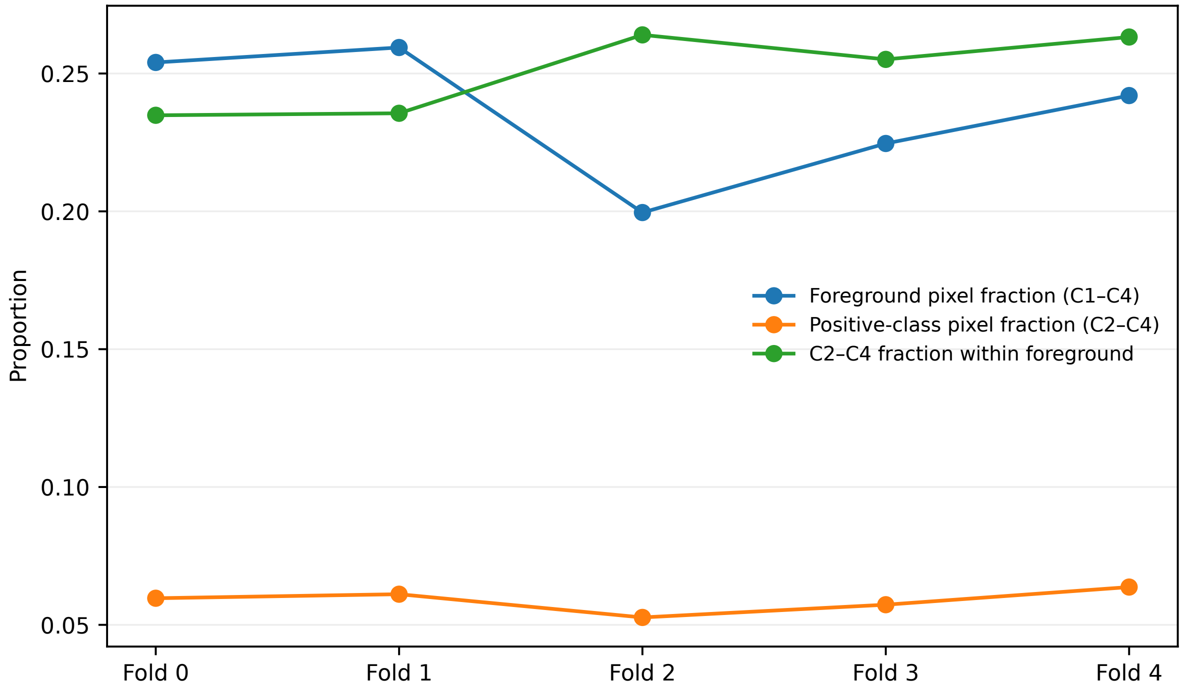

Figure S2: Foreground and positive-intensity balance across the five source-group-preserving outer-test folds. Positive-intensity refers to C2–C4 within the supplied foreground classes.

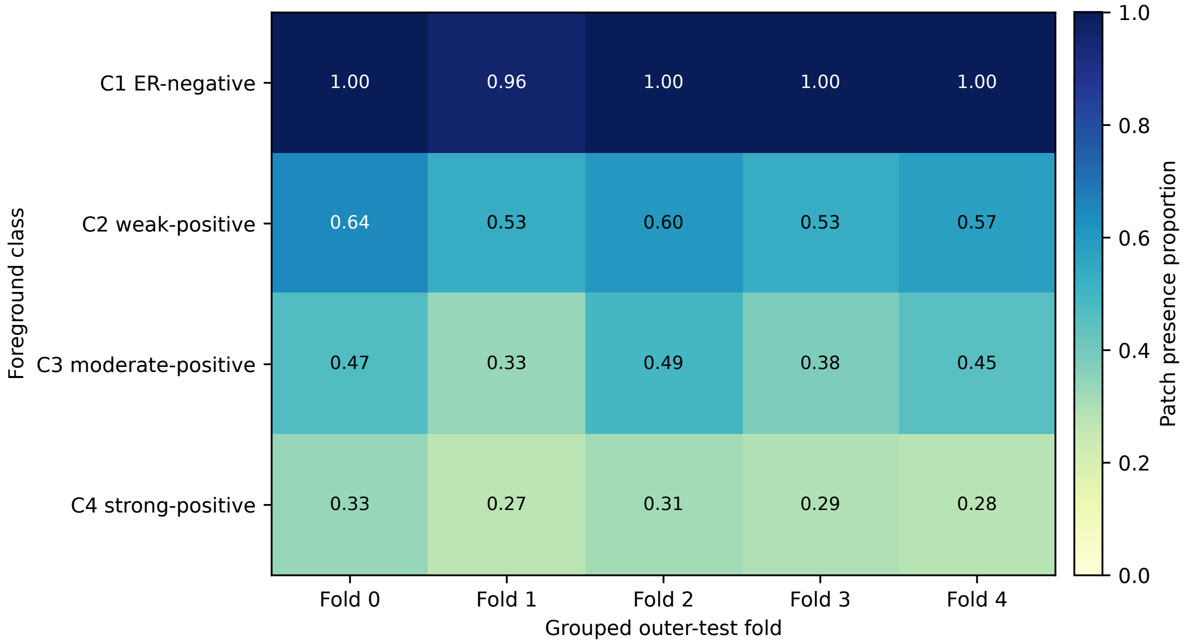

Figure S3: Patch-level class presence across the five source-group-preserving outer-test folds. C2, C3, and C4 were present in every outer fold; exact equality was not imposed because recovered source-group integrity took precedence over patch-level balancing.

Supporting Non-Nested Development Analyses

The figures in this section were produced during the original five-fold model-development stage. The same validation folds were used for checkpoint selection and evaluation in those experiments. They are retained to document architecture exploration, component selection, and sensitivity analysis, but they are not nested outer-test evidence and do not establish final model superiority.

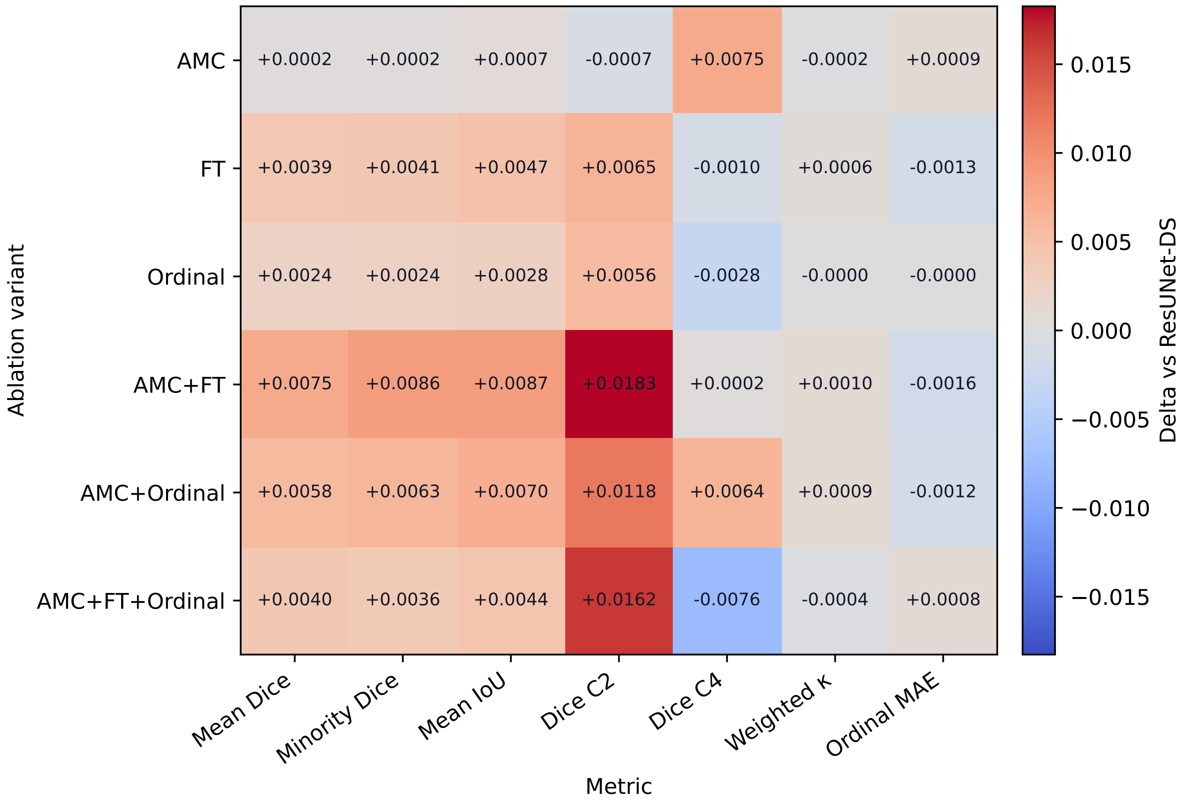

Figure S4: Development-stage component-ablation differences relative to ResUNet-DS. This analysis used the original non-nested training–validation protocol and is retained as supporting model development evidence only.

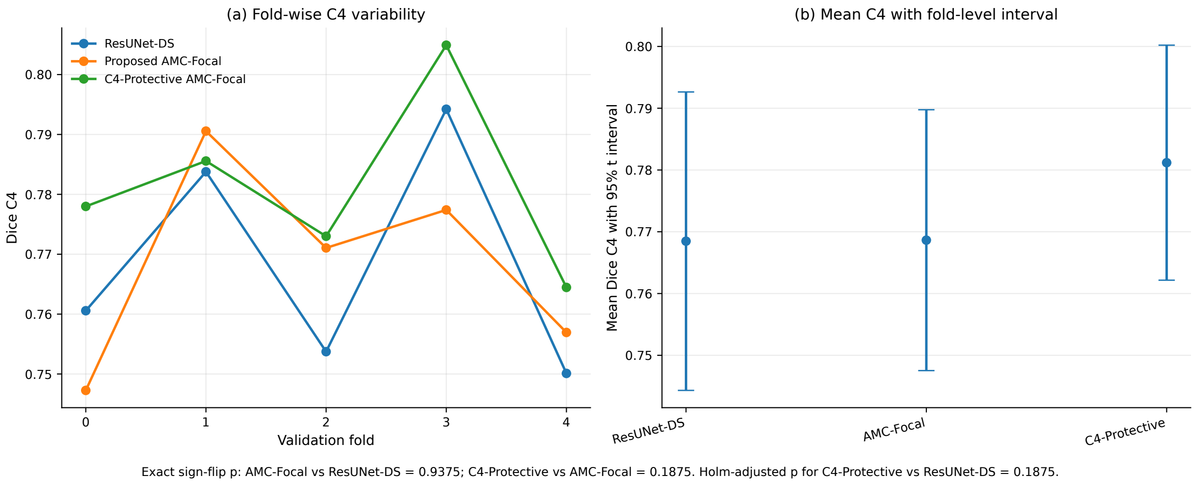

Figure S5: Development-stage comparison of ResUNet-DS, standard AMC-Focal, and C4-protective AMC-Focal. The analysis is exploratory, non-nested, and does not establish a reproducible C2–C4 trade-off.

Nested Post-Hoc Figures

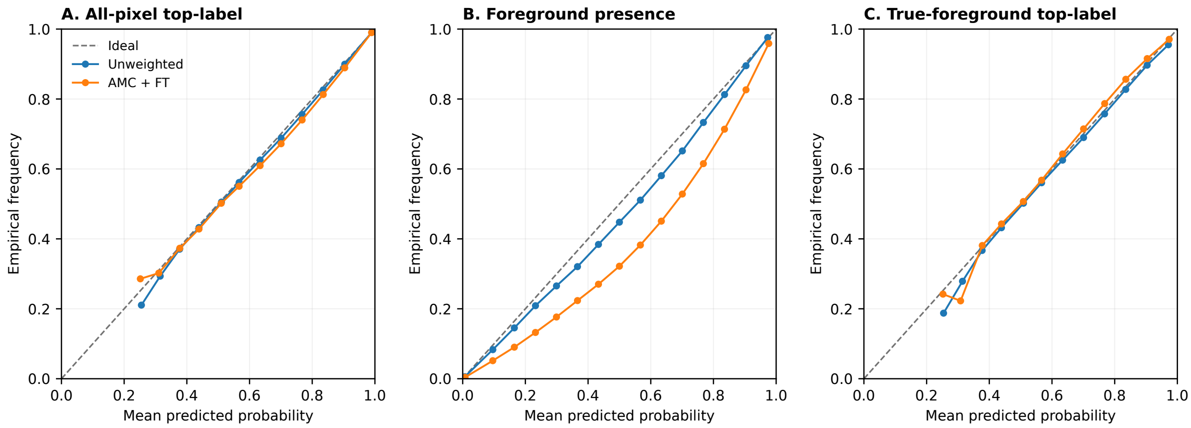

Figure S6: Selected reliability comparison between unweighted random training and weighted adaptive AMC with Focal-Tversky using the frozen source-group-preserving outer-test predictions. Reliability curves are descriptive and do not imply that AMC-Focal was the strongest weighted condition in the final aggregate segmentation comparison.

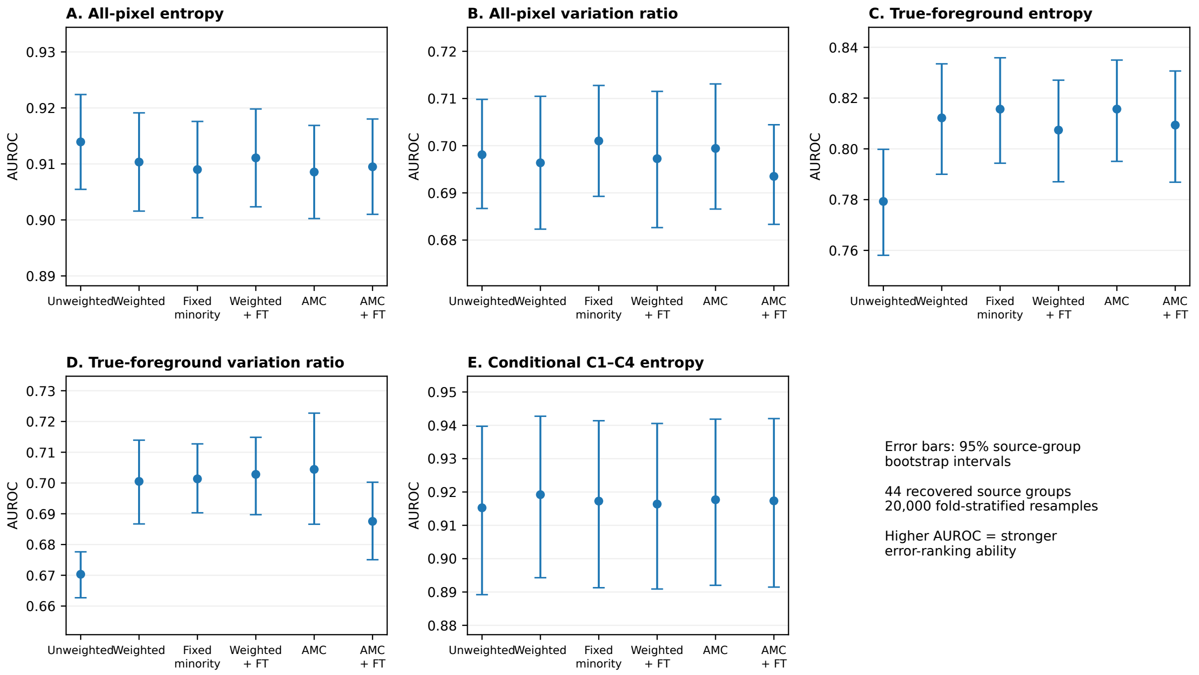

Figure S7: Error-ranking AUROC across all six source-group-preserving conditions for predictive entropy and four-model variation ratio. Higher AUROC indicates better ranking of incorrect pixels above correct pixels; it does not establish calibration, clinical confidence, or reliable automated error detection.

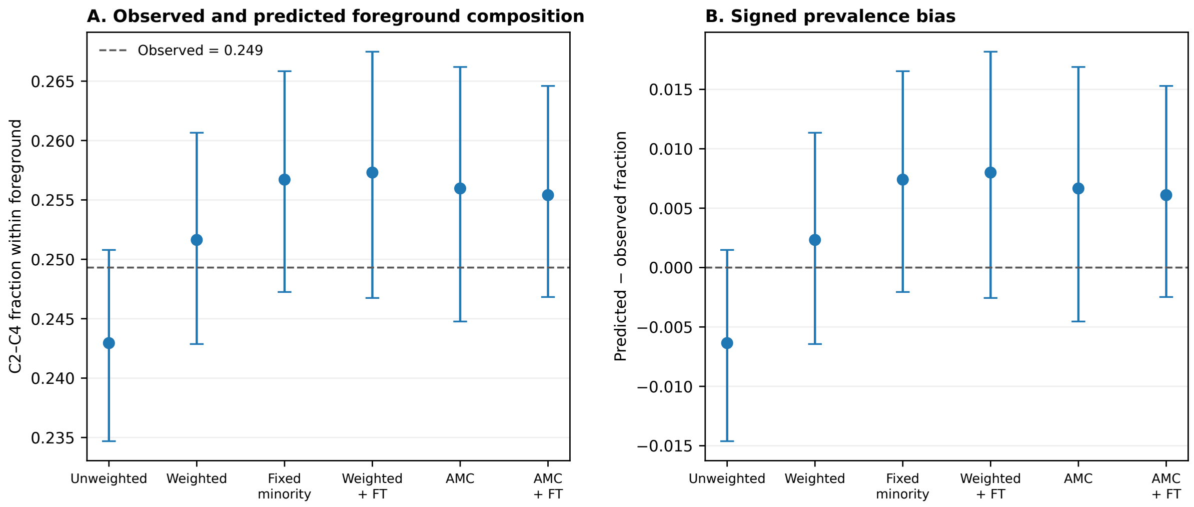

Figure S8: Preservation of the C2–C4 positive-intensity fraction within foreground across the six source-group-preserving conditions. The quantities are pixel-area composition summaries and are not percent-positive tumour-nucleus, Allred, H-score, or slide-level ER measurements.

Supporting Development-Stage Training Curves

The following curves document training behaviour of selected architecture comparators under the original non-nested five-fold development protocol. They are not learning curves from the 120 nested inner-model runs.

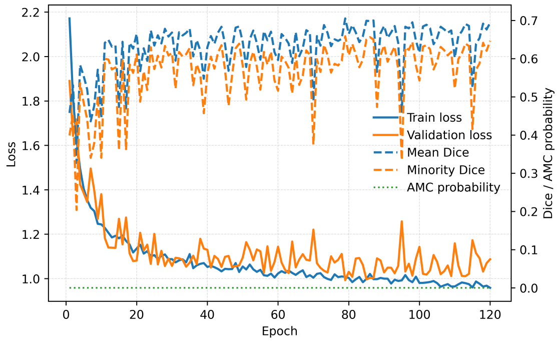

(a) Fold 0

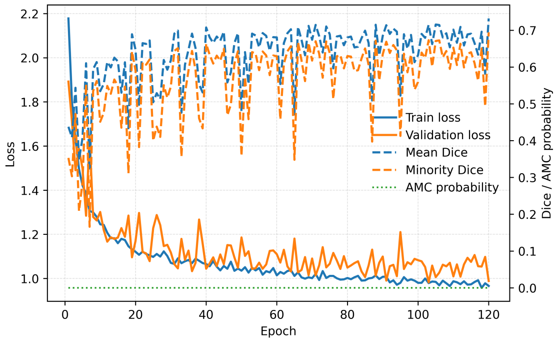

(b) Fold 1

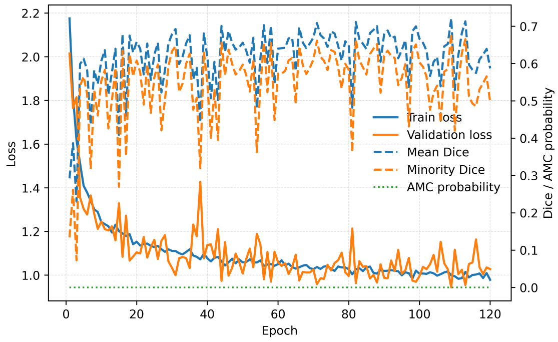

(c) Fold 2

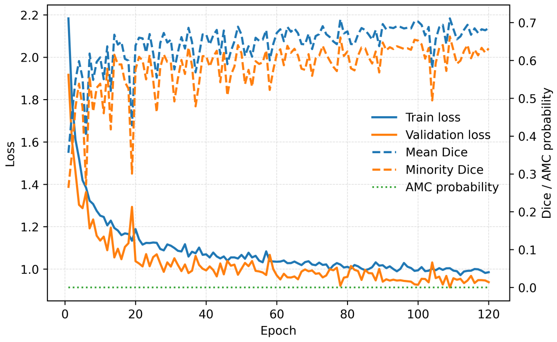

(d) Fold 3

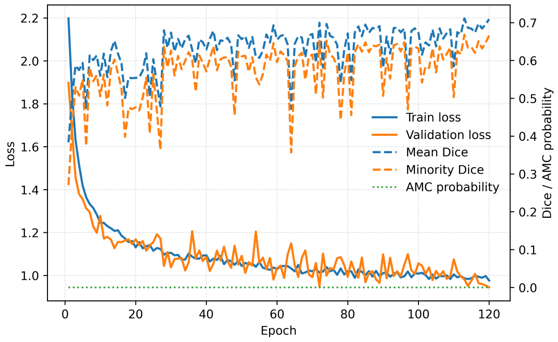

(e) Fold 4

Figure S9: Development-stage Attention U-Net 120 training and validation curves across the original five folds.

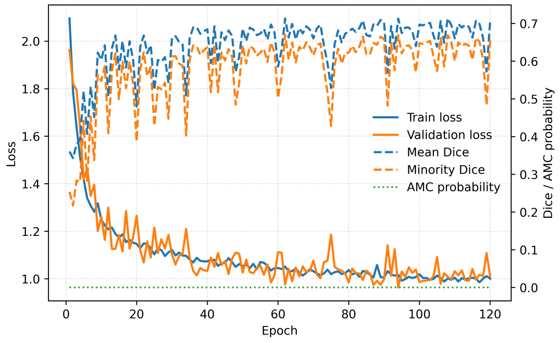

(a) Fold 0

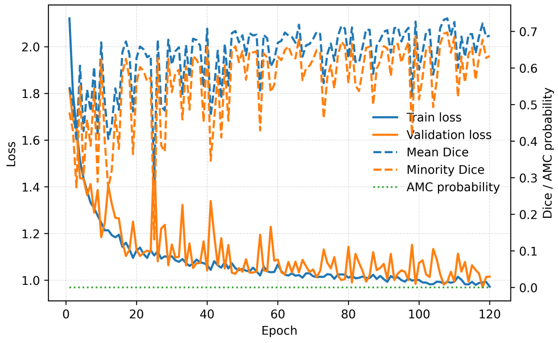

(b) Fold 1

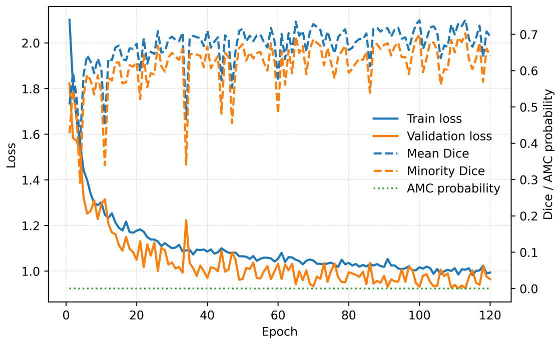

(c) Fold 2

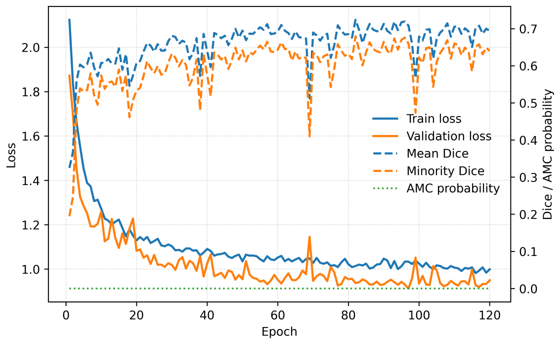

(d) Fold 3

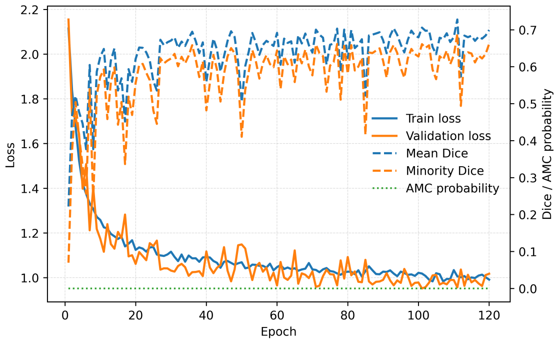

(e) Fold 4

Figure S10: Development-stage TransUNet-style 120 training and validation curves across the original five folds.

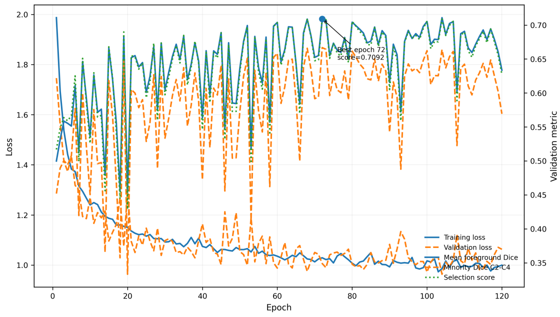

(a) Fold 0

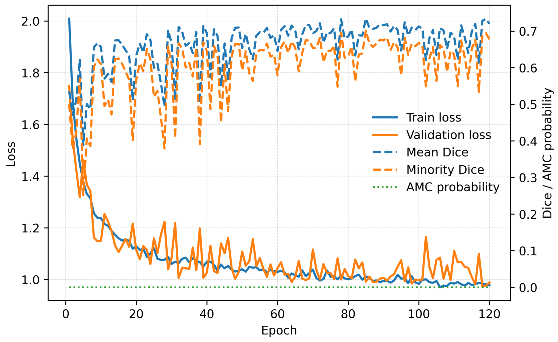

(b) Fold 1

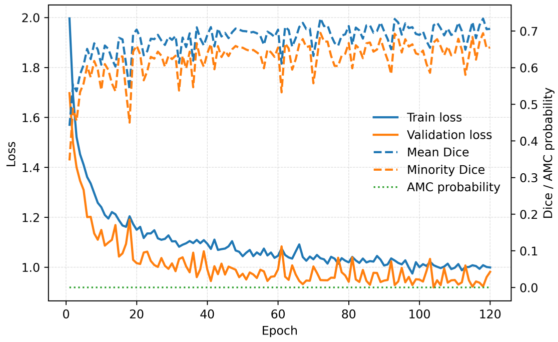

(c) Fold 2

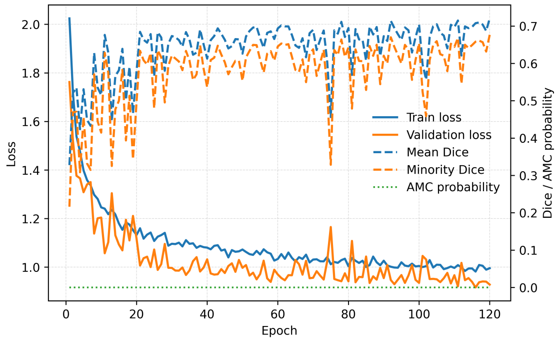

(d) Fold 3

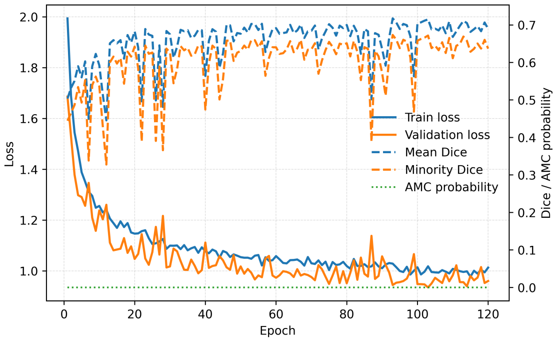

(e) Fold 4

Figure S11: Development-stage U-Net 120 training and validation curves across the original five folds.

Supporting Exploratory Ordinal-Aware Variants

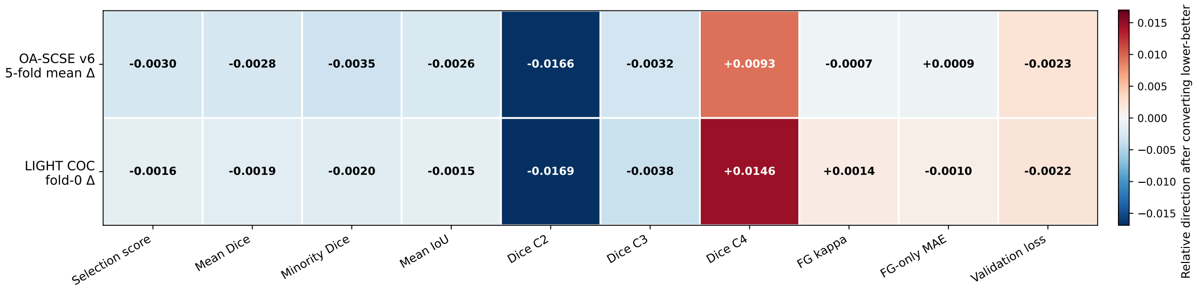

Figure S12: Development-stage ordinal-aware variant trade-off analysis. Values are differences relative to AMC-Focal ResUNet-DS. OA-SCSE v6 reports five-fold development-stage means, whereas LIGHT COC reports fold-0 differences only. Negative foreground ordinal MAE and validation-loss differences indicate improvement. These exploratory variants were not included in the primary nested comparison.

Supporting IHC4BC Expression-Ordering Analysis

The IHC4BC ER-IHC analysis was completed during model development using a previously selected five-model AMC-Focal ResUNet-DS ensemble and was retained as supporting external-domain expression-ordering evidence. It was not repeated across the six final source-group-preserving conditions and was not used to rank those conditions. The prepared subset comprised 1,226 ER-IHC images from seven patients and did not provide compatible pixel-level C1–C4 reference masks.

At image level, predicted C3 area ratio had a Spearman correlation of $\rho = 0.9674$ with strong-DAB ratio. Predicted C2–C4 area ratio was associated with mean DAB ($\rho = 0.9230$) and ER-positive DAB ratio ($\rho = 0.9040$). Within-patient correlations between predicted C3 area and strong-DAB ratio had a median of 0.9518 and ranged from 0.3796 to 0.9806, showing substantial variation among the seven patients. Mean predicted C3 and C4 area ratios were 0.00862 and 0.00037, respectively, and the narrower C4 dynamic range limited its ability to track between-image variation in strong-DAB measurements.

These associations support expression-ordering consistency only. They do not establish equivalence between the internal C1–C4 categories and external DAB-derived variables, direct external segmentation accuracy, Allred or H-score agreement, slide-level ER classification, or patient-level diagnostic performance.

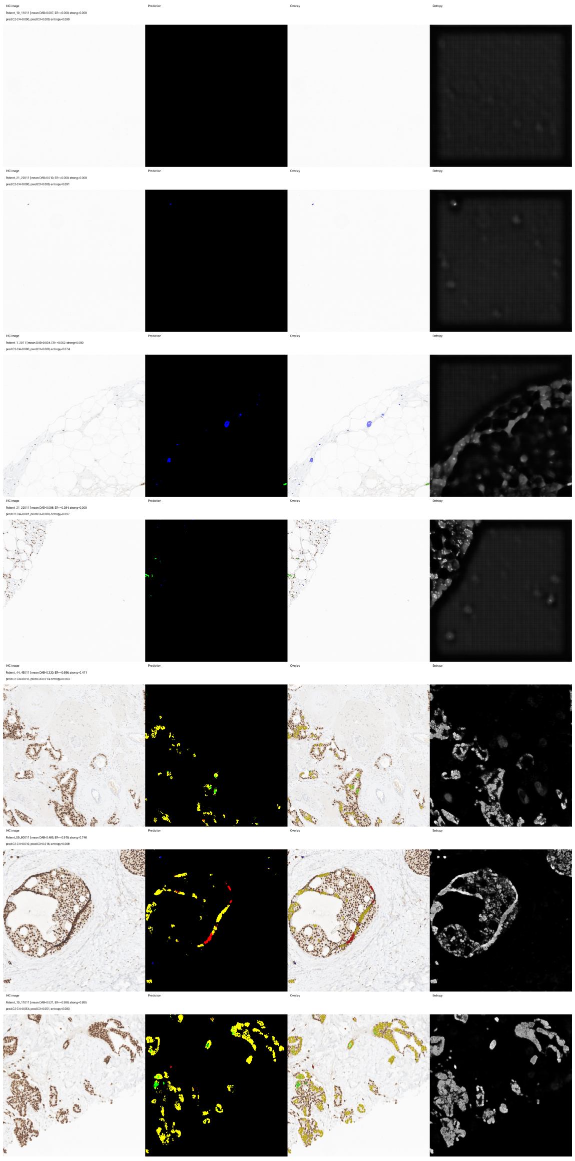

Figure S13: Qualitative montage for the supporting IHC4BC ER-IHC expression-ordering analysis. Representative images are shown across a DAB-expression gradient with model predictions, overlays, and entropy maps. The figure does not constitute direct pixel-level C1–C4 segmentation, class-equivalence, or clinical-score validation.

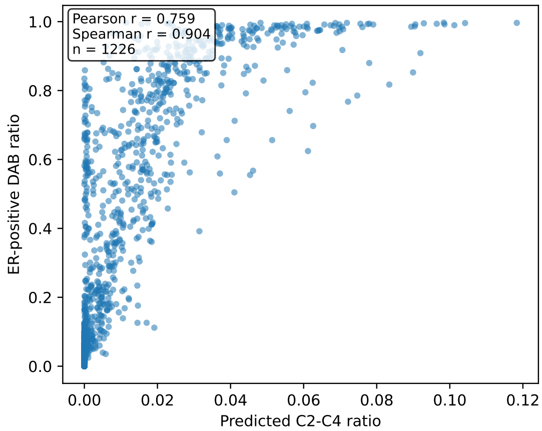

(a) Predicted C2–C4 ratio versus ER-positive DAB ratio.

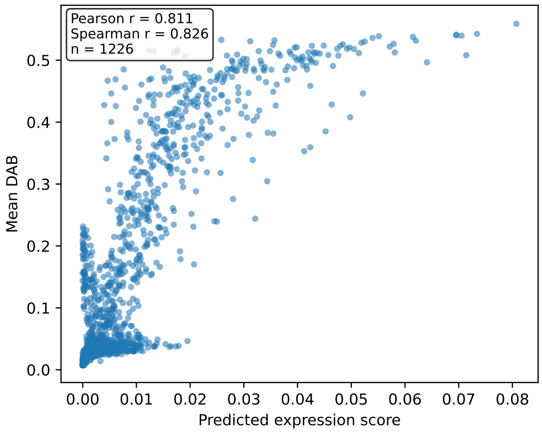

(b) Model-derived expression summary versus mean DAB.

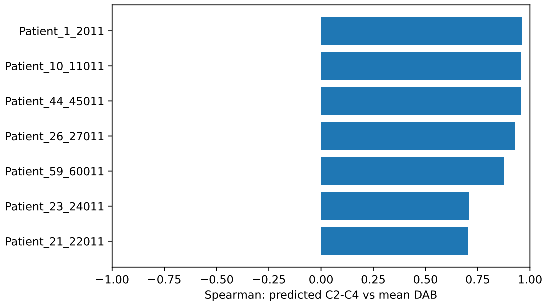

(c) Within-patient C2–C4 ratio versus mean DAB.

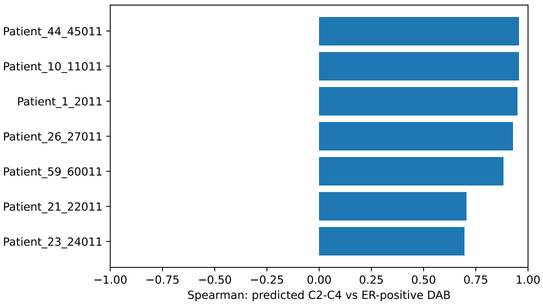

(d) Within-patient C2–C4 ratio versus ER-positive DAB ratio.

Figure S14: Additional IHC4BC expression-ordering analyses. These image-level and within-patient associations do not constitute direct pixel-level C1–C4 segmentation, validated category equivalence, or clinical ER-score validation.

Exploratory HER2-SISH40x Cross-Marker Stress Test

A compact HER2-SISH40x subset was used as an exploratory cross-marker and cross-stain stress test. The subset contained 387 images, comprising 150 Normal, 124 Non-Amplified, and 113 Amplified samples. The previously selected five-model development-fold AMC-Focal ensemble trained on the internal ER-IHC resource was applied without retraining.

The HER2 dataset labels were not mapped to the internal ER-IHC C1–C4 categories because the data represent a different biomarker, staining process, and annotation system. No HER2 classification or scoring metric was calculated. The results describe out-of-domain model behaviour only.

Table S9: Descriptive results from the exploratory HER2-SISH40x cross-marker and cross-stain stress test. These values are not HER2 scores and do not constitute external ER-IHC segmentation validation.

Measure	Normal	Non-Amplified	Amplified
Number of images	150	124	113
Mean predicted foreground ratio	0.0444	0.1279	0.1369
Mean predicted C3 ratio	0.00004	0.00039	0.00083
Mean predicted C4 ratio	0.00026	0.00106	0.00150
Mean normalised predictive entropy	0.0589	0.0917	0.1197
Mean maximum probability	0.9649	0.9375	0.9178

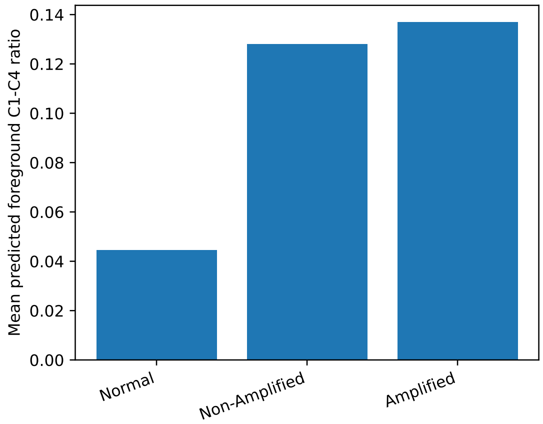

(a) Predicted foreground ratio.

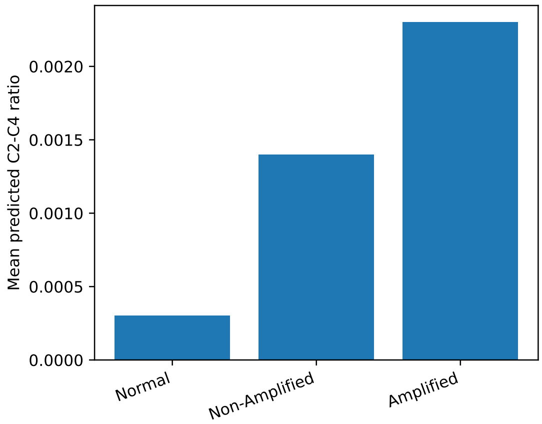

(b) Predicted C2–C4 ratio.

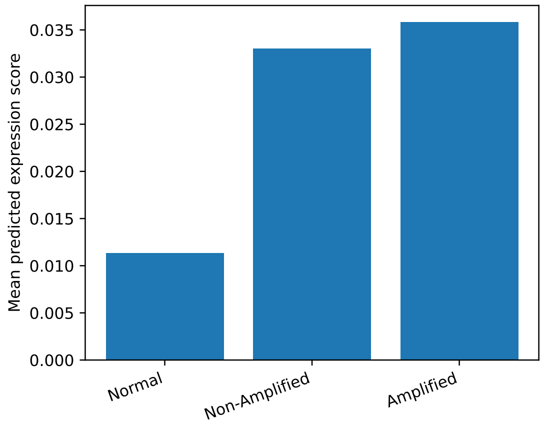

(c) Predicted expression score.

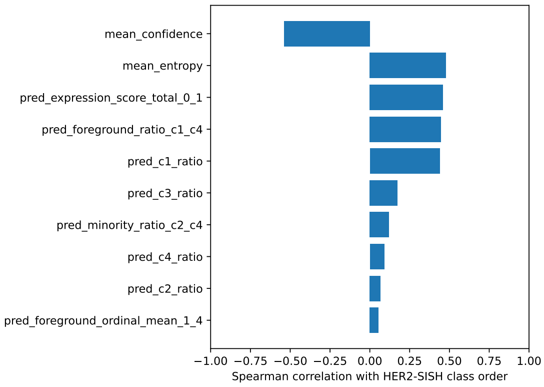

(d) Exploratory associations across the three HER2 source-label categories.

Figure S15: Exploratory HER2-SISH40x cross-marker and cross-stain stress test. These outputs are descriptive out-of-domain ER-IHC model summaries, not HER2 predictions or HER2 scores, direct ER-IHC C1–C4 validation, or evidence of class-level transfer.

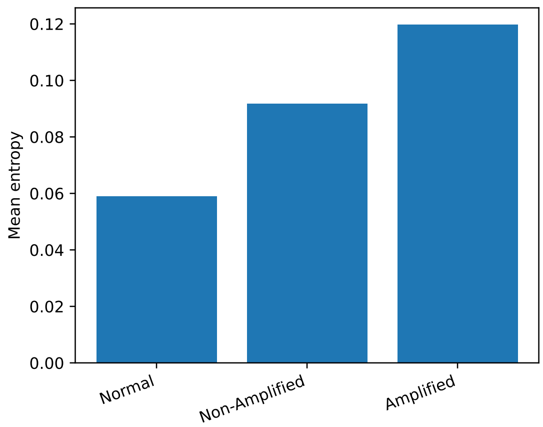

(a) Mean entropy across HER2 source-label categories.

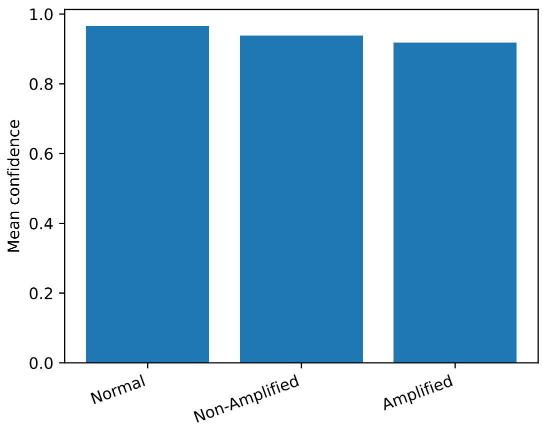

(b) Mean maximum probability across HER2 source-label categories.

Figure S16: Entropy and maximum-probability behaviour in the exploratory HER2-SISH40x stress test. These quantities are post-hoc out-of-domain indicators and not calibrated clinical confidence estimates.

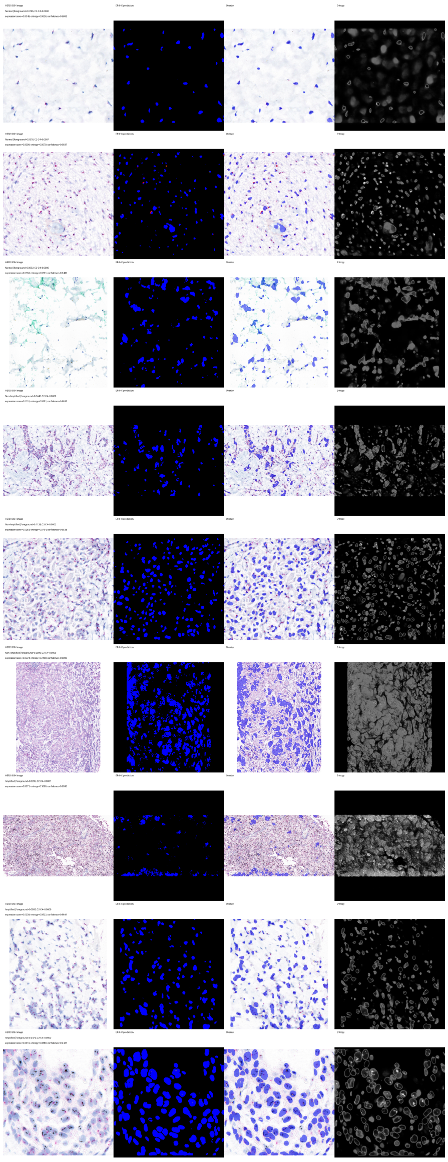

Figure S17: Qualitative montage for the exploratory HER2-SISH40x cross-marker and cross-stain stress test. The figure illustrates out-of-domain behaviour only and should not be interpreted as HER2 scoring, external ER-IHC validation, or transfer of the internal C1–C4 categories.

Additional Nested and Supporting Analyses

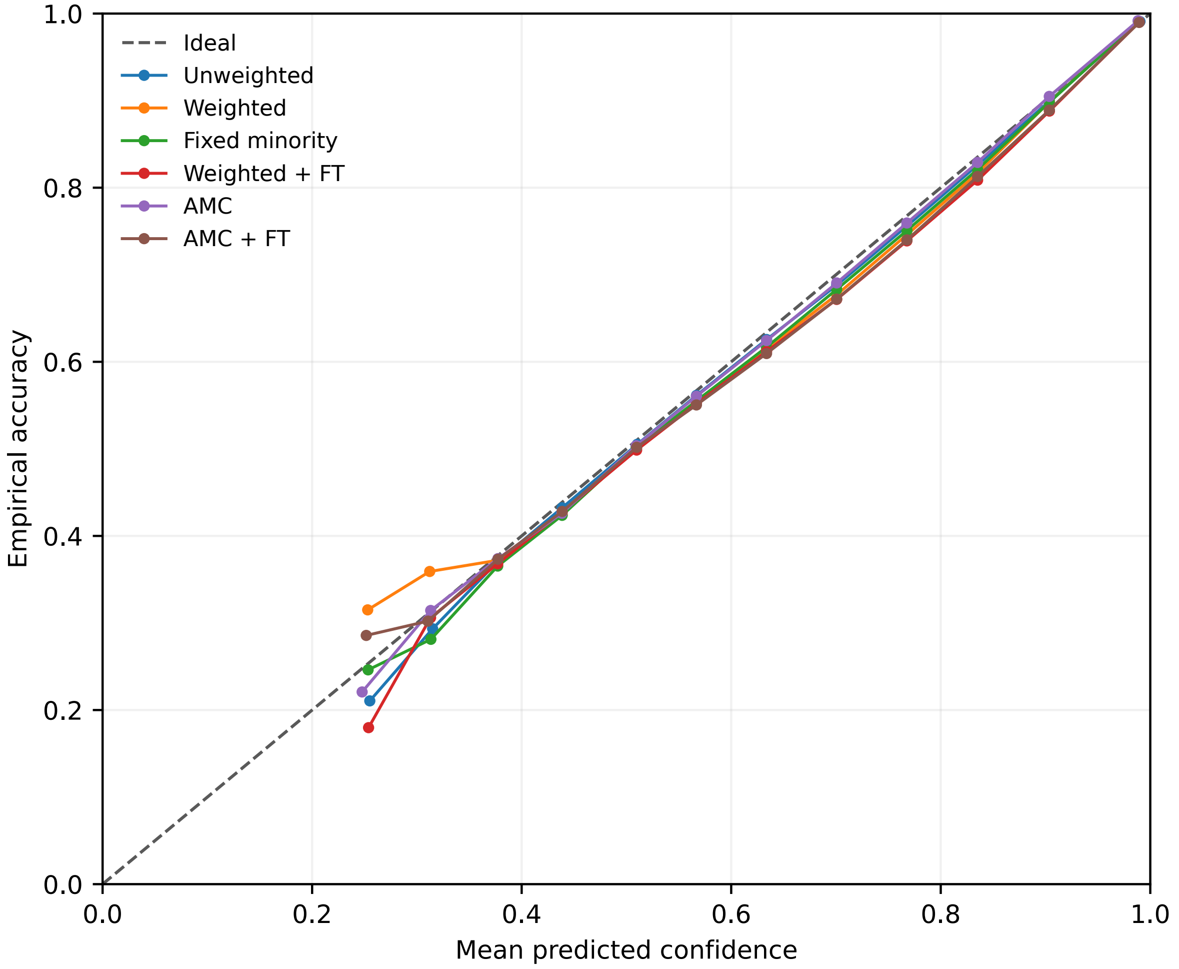

Figure S18: All-condition top-label reliability from the frozen source-group-preserving outer-test predictions. Reliability curves are descriptive and ECE remains bin-dependent; no pixel-level inferential claim is made from these curves.

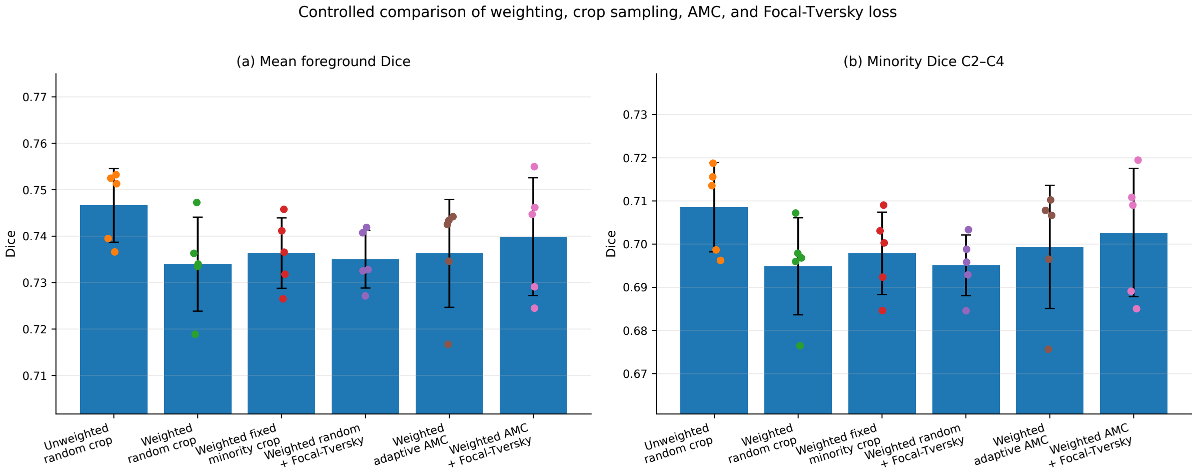

Figure S19: Supporting development-stage controlled comparison of weighting, sampling, AMC, and Focal-Tversky strategies under the earlier non-nested protocol. This figure documents model development only and is not part of the primary source-group-preserving outer-test comparison.

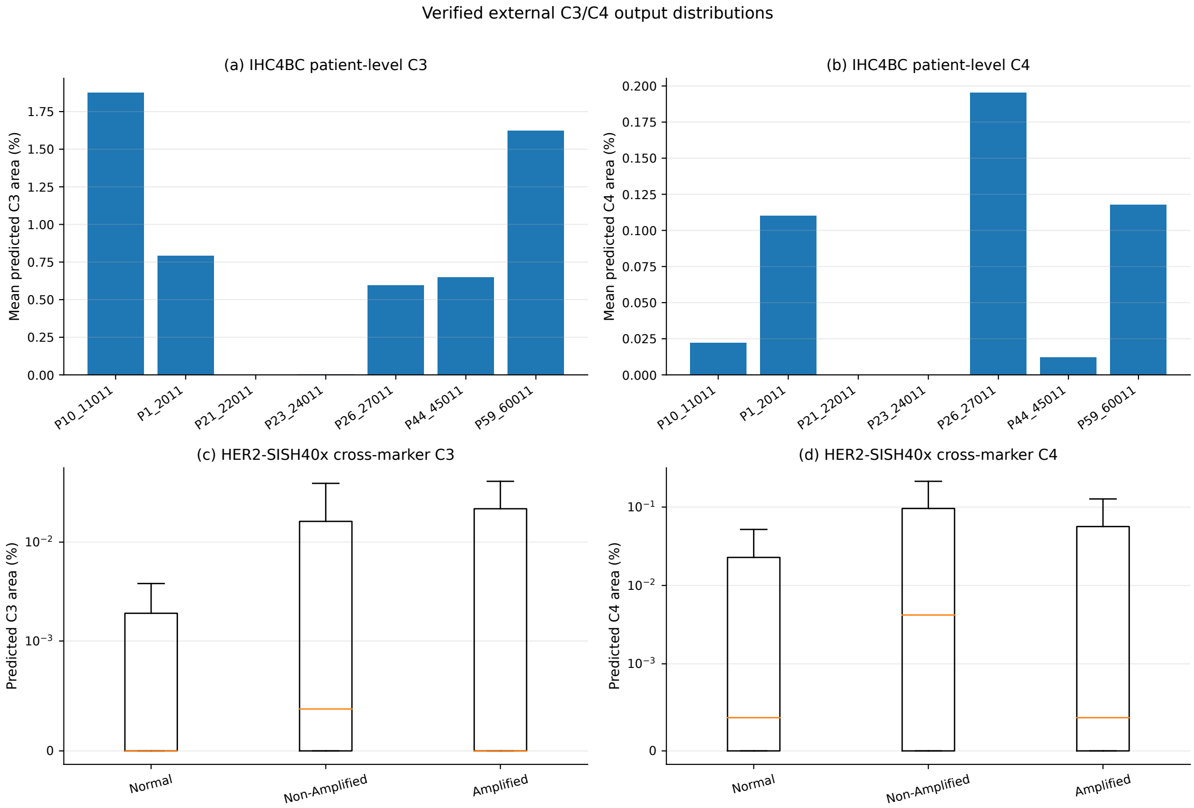

Figure S20: External distributions of predicted C3 and C4 fractions. The IHC4BC panel supports expression-ordering interpretation, whereas the HER2-SISH40x panel describes exploratory cross-marker and cross-stain behaviour. Neither constitutes direct validation against compatible C1–C4 reference masks, clinical ER-score validation, or HER2 scoring.

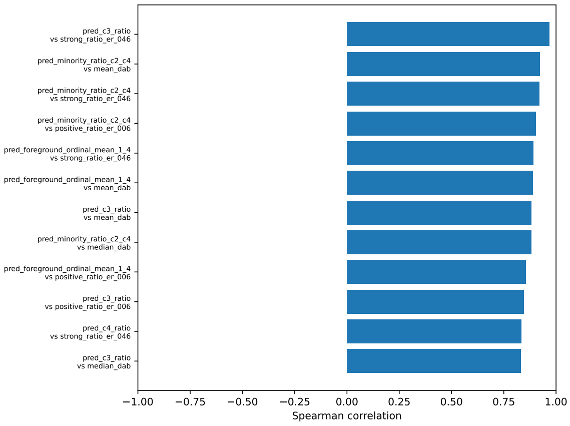

(a) Highest image-level Spearman associations.

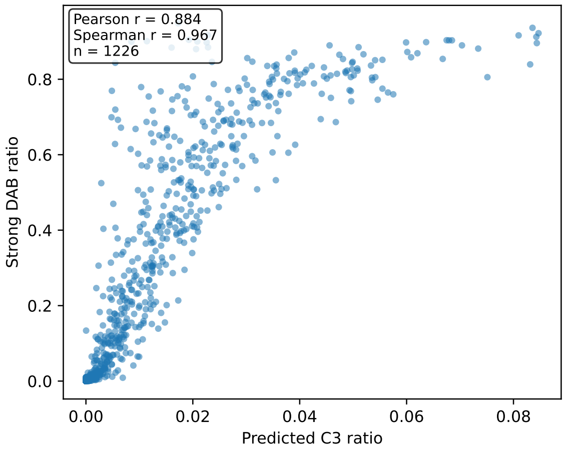

(b) Predicted C3 ratio versus strong-DAB ratio.

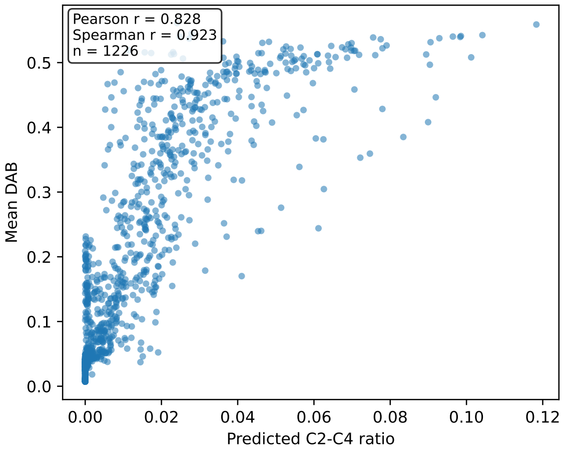

(c) Predicted C2–C4 ratio versus mean DAB.

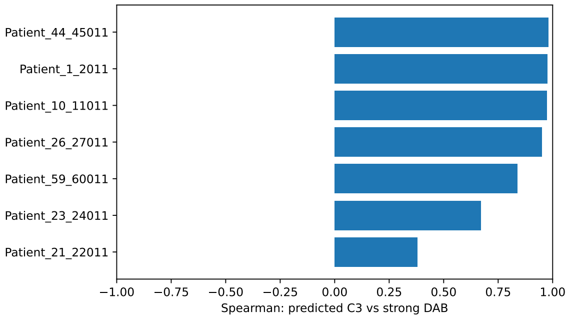

(d) Within-patient C3-versus-strong-DAB associations.

Figure S21: Principal supporting IHC4BC expression-ordering results. The panels show image-level and within-patient associations between model-derived spatial summaries and DAB-derived variables. These associations are not direct external segmentation validation, category-equivalence validation, or clinical-score validation.
